# Identification and Single-Cell Functional Characterization of an Endodermally Biased Pluripotent Substate in Human Embryonic Stem Cells

**DOI:** 10.1016/j.stemcr.2018.04.015

**Published:** 2018-05-17

**Authors:** Thomas F. Allison, Andrew J.H. Smith, Konstantinos Anastassiadis, Jackie Sloane-Stanley, Veronica Biga, Dylan Stavish, James Hackland, Shan Sabri, Justin Langerman, Mark Jones, Kathrin Plath, Daniel Coca, Ivana Barbaric, Paul Gokhale, Peter W. Andrews

**Affiliations:** 1 Centre for Stem Cell Biology, Department of Biomedical Science, University of Sheffield, Sheffield S10 2TN, UK; 2 Signal Processing and Complex Systems Group, Department of Automatic Control and Systems Engineering, University of Sheffield, Sheffield S10 2TN, UK; 3 MRC Centre for Regenerative Medicine, Institute for Stem Cell Research, School of Biological Sciences, University of Edinburgh, Edinburgh EH16 4UU, UK; 4 Stem Cell Engineering, Biotechnology Center, Technische Universitaet Dresden, 01307 Dresden, Germany; 5 MRC Molecular Haematology Unit, MRC Weatherall Institute of Molecular Medicine, Radcliffe Department of Medicine, University of Oxford, John Radcliffe Hospital, Oxford OX3 9DS, UK; 6 UCLA School of Medicine, Department of Biological Chemistry, University of California Los Angeles, Los Angeles, CA, USA; 7 School of Medicine, Faculty of Biology and Health, University of Manchester, Manchester M13 9PT, UK

**Keywords:** GATA6, lineage priming, human embryonic stem cell heterogeneity, differentiation bias

## Abstract

Human embryonic stem cells (hESCs) display substantial heterogeneity in gene expression, implying the existence of discrete substates within the stem cell compartment. To determine whether these substates impact fate decisions of hESCs we used a GFP reporter line to investigate the properties of fractions of putative undifferentiated cells defined by their differential expression of the endoderm transcription factor, GATA6, together with the hESC surface marker, SSEA3. By single-cell cloning, we confirmed that substates characterized by expression of *GATA6* and SSEA3 include pluripotent stem cells capable of long-term self-renewal. When clonal stem cell colonies were formed from *GATA6*-positive and *GATA6*-negative cells, more of those derived from *GATA6*-positive cells contained spontaneously differentiated endoderm cells than similar colonies derived from the *GATA6*-negative cells. We characterized these discrete cellular states using single-cell transcriptomic analysis, identifying a potential role for SOX17 in the establishment of the endoderm-biased stem cell state.

## Highlights

• Subsets of hESCs can co-express pluripotency-associated and lineage-specific genes
• hESCs co-expressing *GATA6* are capable of long-term self-renewal
• Single *GATA6*-expressing hESCs regenerate *GATA6*-negative cells
• *GATA6*-expressing hESCs are biased in their propensity for differentiation

## Graphical Abstract

**Figure:**
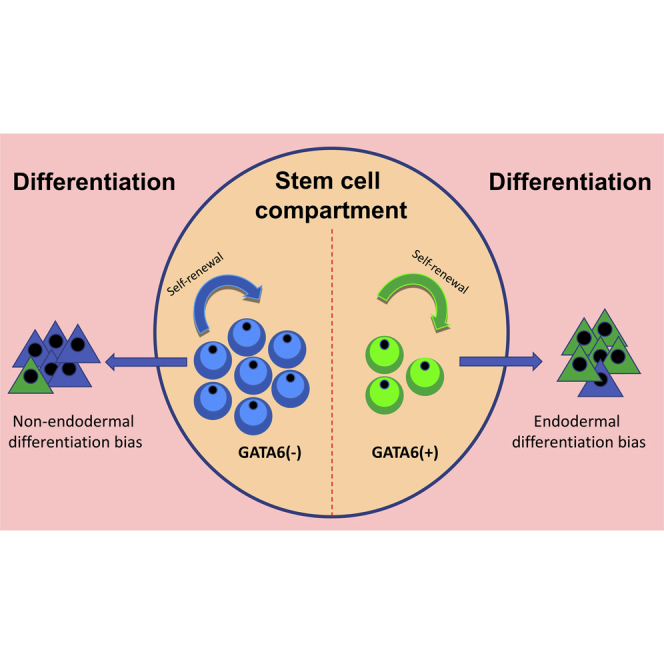

## Teaser

Human embryonic stem cells have the capacity to turn into any cell type within the adult. Peter Andrews and colleagues have shown that subtle differences between individual cells can functionally bias the resulting cell type that is produced. Generating and purifying these biased cells may therefore improve the derivation of medically relevant cell types.

## Introduction

Human embryonic stem cells (hESCs) offer opportunities for a wide range of applications in human health care, provided that effective methods are developed for controlling their differentiation. A central problem for stem cell biology, whether for pluripotent stem cells from the early embryo, or multipotent stem cells from later tissues, is to establish how such cells make fate decisions between self-renewal or differentiation and then how they choose between alternative pathways of differentiation (Murry and Keller, 2008). In part, the decision any individual stem cell makes depends upon external cues, and many studies focus on the response of stem cells to particular signals, whether diffusible cytokines, the extracellular matrix or cell:cell interactions (Semrau and van Oudenaarden, 2015). However, as cell characterization has become more refined and single-cell analyses have become feasible, many studies have also highlighted the heterogeneity of stem cell populations, making it possible to cluster cells into different subsets (Hough et al., 2009, Hough et al., 2014). This raises the question of whether this heterogeneity is “noise” with no relevance to fate decisions, or whether the different subsets of stem cells respond differently to external cues so that their ultimate fate depends on a combination of extrinsic and intrinsic factors. By definition, stem cells assigned to different subsets must all be capable of self-renewal and the same range of differentiation, but it is possible that the different subsets correspond to different, interconvertible substates in which the stem cells exhibit distinct properties (Arias and Brickman, 2011, Draper et al., 2002, Enver et al., 2005, Enver et al., 2009).

Among hematopoietic stem cells, heterogeneity in the patterns of gene expression at the single-cell level has been used to suggest the existence of multi-lineage priming, whereby subsets of stem cells activate components of different lineage-related regulatory genes prior to commitment to differentiate (Hu et al., 1997, Huang et al., 2007). Further, different subsets of a myeloid progenitor cell separated by differential surface markers appeared to have different propensities for monocyte and erythroid differentiation, although both were capable of self-renewal (Chang et al., 2008). However, in another study based on single-cell analyses (Pina et al., 2012), that conclusion was questioned since the apparent lineage-biased subsets could themselves be further subdivided into self-renewing and lineage-committed cells, emphasizing the need for clonal analyses to confirm the co-existence of self-renewal capacity and lineage bias in a single cell. In the pluripotent context, interconvertible subsets of mouse embryonic stem cells have been identified using reporters for stem cell-associated transcription factors such as NANOG (Chambers et al., 2007), STELLA (Hayashi et al., 2008), or REX1 (Toyooka et al., 2008), or lineage-associated transcription factors such as HEX (Canham et al., 2010), and shown to exhibit different functional properties.

We previously identified a transitory state of hESCs, marked by lack of the surface marker SSEA3, with an apparently greater tendency to differentiate (Enver et al., 2005), while Laslett et al. (2007) reported a gradation in expression of the surface markers CD9 and GCTM2 as hESCs transited from an undifferentiated to differentiated state (Laslett et al., 2007). However, although these observations indicate substates with a greater or lesser tendency to differentiate, it is unclear whether substates can be identified with different biases with respect to the lineages they follow after differentiation. Previously, we inferred the existence of such lineage-biased substates in the pluripotent human embryonal carcinoma cell line NTERA2, but could not specifically identify the biased cells prior to differentiation (Tonge et al., 2010).

In a recent study of gene expression in individual hESCs, we observed that among cells expressing characteristic features of undifferentiated cells, notably the surface antigen SSEA3, and the transcription factors OCT4 and NANOG, some also expressed genes typically associated with endoderm differentiation, such as *GATA6* (Gokhale et al., 2015). To test whether these cells are functional, self-renewing stem cells, we have produced and analyzed an hESC line, Shef4, carrying a GFP reporter knocked into the *GATA6* locus by gene targeting, as a tool to interrogate whether functionally biased substates exist within the over-arching pluripotent stem cell state. We have found that the undifferentiated cells can not only interconvert between substates that do and do not express *GATA6*, but also that in the *GATA6-*expressing substate they have a higher probability of endoderm differentiation.

## Results

### A *GATA6-GFP* Reporter Cell Line Reveals Orders of hESC Heterogeneity

To investigate the dynamics of *GATA6* expression in live hESCs, we generated a Shef4 hESC line (Aflatoonian et al., 2010) with an GFP reporter knockin into one allele of the *GATA6* locus by Zinc Finger Nuclease-mediated homologous recombination. The GFP reporter knockin into the translational initiation codon of the *GATA6* locus was designed to express GFP under the control of the endogenous *GATA6* promoter (Figure S1A). Shef4 clones with gene targeted integrations by homologous recombination were identified, and one heterozygous knockin clone (S4G6 4/F-9) was confirmed to contain a single insertion of the GFP reporter at the *GATA6* locus with no additional integrations (Figure S1B). This clone was further genetically modified to delete the neomycin resistance gene selection cassette by recombinase-mediated excision (Supplemental Experimental Procedures), and a resulting clone (S4G6 A3) was generated with the expected DNA rearrangement (Figure S1B) and a normal XY karyotype (Figure S1C). To validate the fidelity of the reporter line, we differentiated both the parental Shef4 cells and the reporter cell line S4G6 A3 toward endoderm. As expected, the Shef4 cells showed increased GATA6 protein, but no GFP expression, whereas the reporter line showed an increase in GFP expression and GATA6 protein in a correlative manner as anticipated for the above knockin strategy (Figure S1D). To assess whether the knockin of the GFP cassette into the *GATA6* locus altered endodermal differentiation capacity, we performed qPCR for genes characteristic of endoderm/primitive streak. Gene expression levels were found to be similar between the parental Shef4 cells and the GFP knockin line, confirming the differentiation capacity of the reporter line (Figure S1E). Additionally, we investigated whether the insertion of GFP into the *GATA6* locus altered the *GATA6* RNA level in the hESC state. We found by performing qPCR a slightly reduced level of *GATA6* expression in the reporter knockin line relative to the Shef4 parental cells qualitatively consistent with the expectation that the reporter integration should result in premature termination of *GATA6* transcription (Figure S1F).

**Figure 1:**
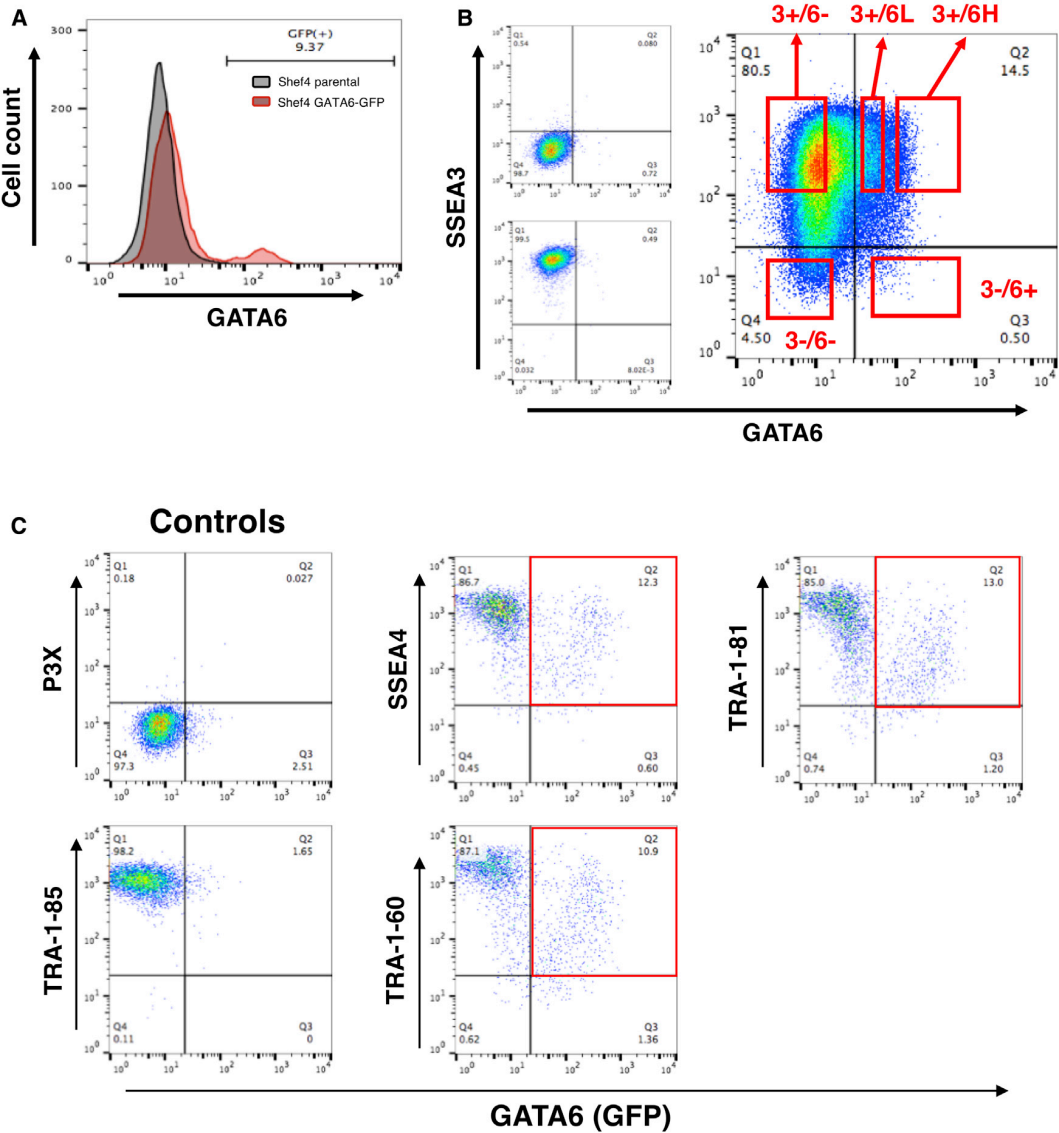
*GATA6* Is Expressed in a Small Subset of hESCs (A) Representative FACS plot of the Shef4 *GATA6*-GFP reporter line S4G6 A3 cultured in KO/SR and MEF conditions. Black peak represents the unmodified parental Shef4 control line, and red, the Shef4 *GATA6-*GFP reporter line. (B) Representative FACS plot of SSEA3 vs *GATA6* expression. Left panels show gating controls P3X (above) and TRA-1-85 (below) on the Shef4 parental line. Right panel shows the identification of distinct cell populations: SSEA3 high, *GATA6* negative (3+/6−); SSEA3 high, *GATA6* low (3+/6L); SSEA3 high, *GATA6* high (3+/6H); SSEA3 negative *GATA6* high (3−/6+), of the *GATA6* reporter line. (C) Representative FACS plots of additional stem cell surface markers, SSEA3, TRA-1-81 or SSEA4 vs *GATA6* expression with the same controls as (B).

Having validated our reporter line, we subsequently used expression of GFP as a measure of the *GATA6* transcriptional state, which we refer to throughout the manuscript as *GATA6*. By flow cytometry, we observed that the reporter line grown in KO/SR (Knockout DMEM and 20% Knockout Serum Replacement) on mouse embryo fibroblast (MEF) feeders, contained a subset of 2%–10% cells expressing *GATA6* (Figure 1A). We also found varying degrees of *GATA6* expression denoted by “low” and “high.” To determine whether GFP expression correlated with GATA6 protein expression in self-renewing conditions, we stained the reporter line in self-renewal conditions with a GATA6 antibody and found that as GFP intensity increased, the levels of GATA6 protein also increased (Figure S2A). To begin characterizing *GATA6* expressing cells, we first tested whether they expressed SSEA3, a sensitive cell surface marker that we have used extensively to identify undifferentiated hESCs (Andrews et al., 1982, Enver et al., 2005, Gokhale et al., 2015). We found a new level of cellular heterogeneity and the appearance of distinct populations of hESCs in culture. The most apparent population expressed high levels of SSEA3 with no *GATA6* expression (3+/6−), with smaller populations expressing high *GATA6* levels with no SSEA3 (3−/6+), and no SSEA3 or *GATA6* (3−/6−). Notably, we saw co-expressing populations consisting of high SSEA3 with low *GATA6* (3+/6L) and high SSEA3 with high *GATA6* (3+/6H) expression (Figure 1B). To determine whether this co-expression was a feature of just SSEA3, we also examined three other stem cell-associated surface antigens, SSEA4, TRA-1-60, and TRA-1-81 (Adewumi et al., 2007). Similar to SSEA3, these three antigens showed co-expression with *GATA6* (Figure 1C). These results suggest that hESCs exist within substates demarcated by the expression of stem cell surface markers and GATA6, a transcription factor usually associated with endoderm differentiation. This then raised the question of whether GATA6 confers a bias when these cells differentiate.

### *GATA6-*Expressing Cells Have Gene Expression Patterns Indicative of Early Endoderm Differentiation

**Figure 2:**
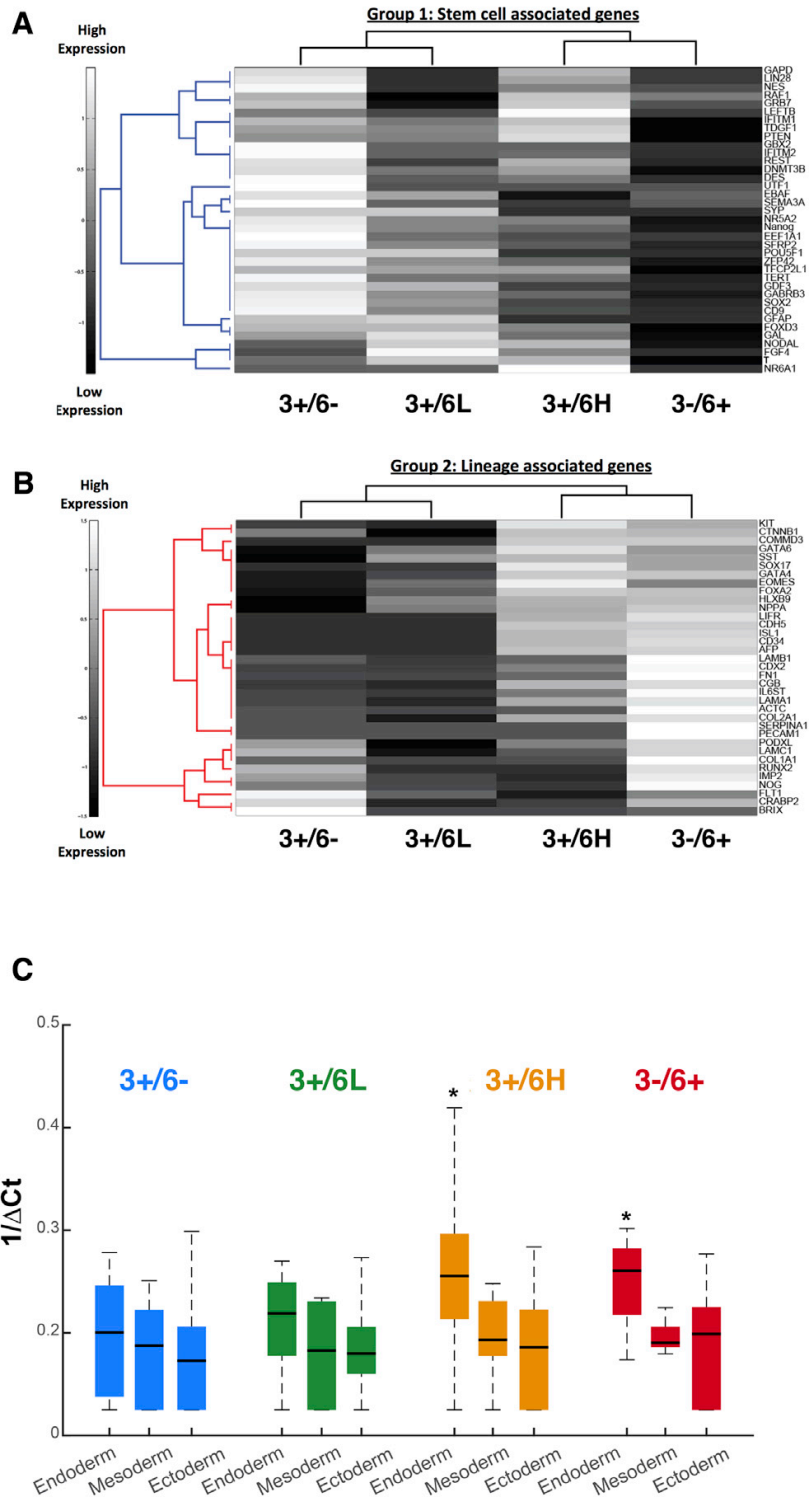
Gene Expression Profiles of Fractions 3+/6−, 3+/6L, 3+/6H, and 3−/6+ (A and B) qPCR using an Applied Biosystems pluripotency TaqMan array on each cell fraction. Hierarchical clustering using Spearman's rank correlation showed strong segregation of genes into two groups: stem cell-associated (group 1) (A) and lineage-associated genes (group 2) (B) with respective gene names. Colormap indicates level of expression of 1/Δ-CT values standardized by row. (C) Boxplot analysis of average gene expression of lineage-specific genes in each cell fraction grouped by specific germ layer. ^∗^Kruskal-Wallis statistical test results are indicated for p values <0.05.

To better understand the gene expression differences between the cellular substates we identified, we performed qPCR on the four cell fractions (Figure 1B) using the TaqMan Low Density Pluripotency Array (Adewumi et al., 2007). Hierarchical analysis revealed two major clusters: one cluster, mostly comprising stem cell-related genes, was expressed in the 3+/6− cells, and downregulated in the 3−/6+ subset, whereas a second cluster, mostly comprising various differentiation-related genes, showed the opposite pattern. The 3+/6L and 3+/6H subsets showed intermediate patterns of expression, which could be interpreted to represent intermediate stages in a progression from the 3+/6− state to the 3−/6+ state (Figures 2A and 2B). The changes in expression of a few genes, e.g., *LIN28*, *GRB7*, *NR6A,* and *T*, did not fit this simple progressive view, but most likely this reflects the complexities and persistent heterogeneity of the cell subsets (Figures 2A and S2B). When genes associated *a priori* with endoderm, mesoderm, and ectoderm differentiation were grouped (Adewumi et al., 2007), we found no overall difference between the subsets with respect to mesoderm and ectoderm-related genes, but there was a significant increase in expression of genes associated with endoderm in the 3+/6H and 3−/6+ subsets (Figure 2C). Therefore, *GATA6* expression appeared to be correlated with a reduction in stem cell-associated genes and was coincident with an increase in, specifically, endodermal gene expression.

### A Subset of *GATA6*-Expressing Cells Maintain Pluripotency

**Figure 3:**
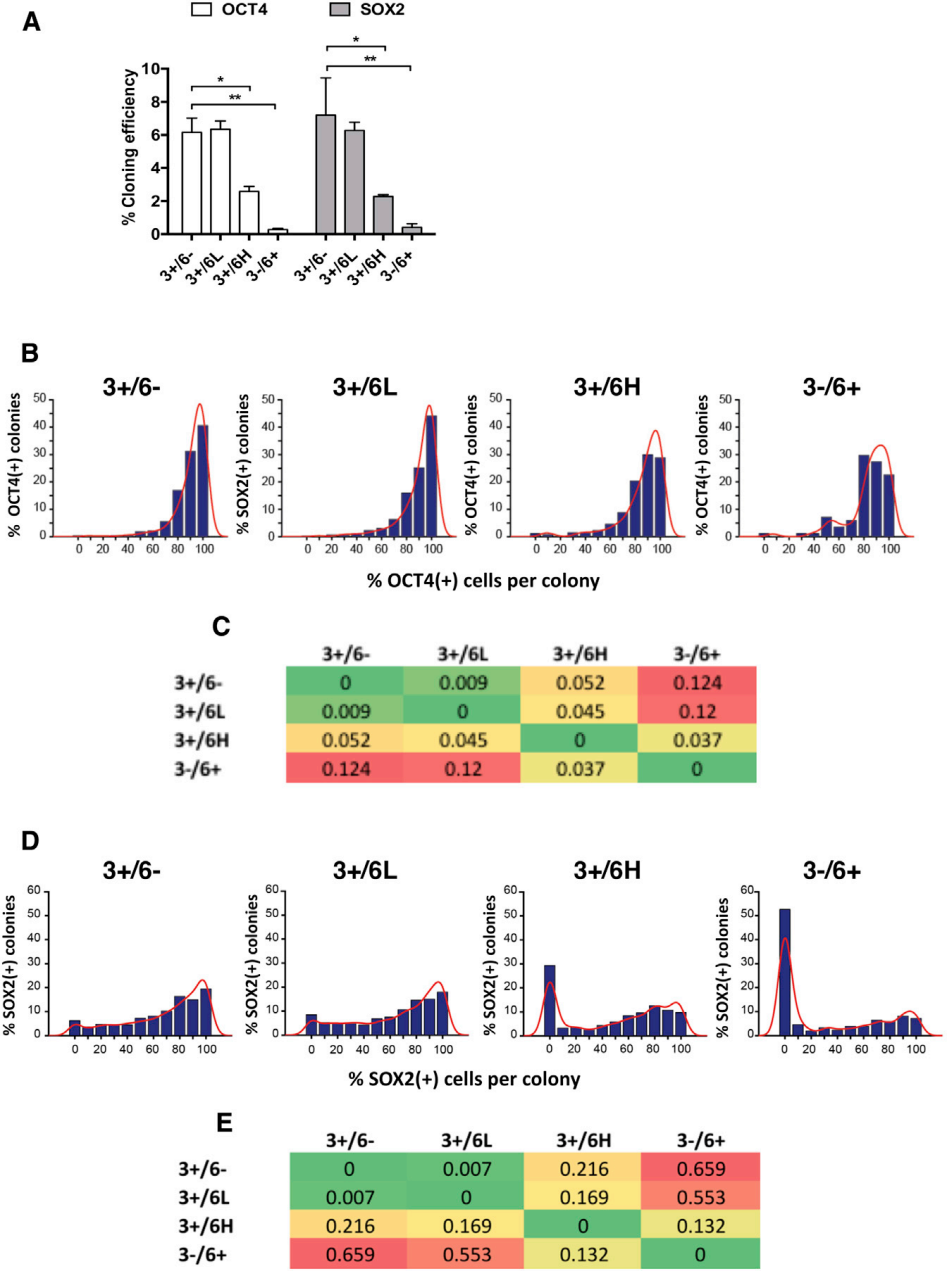
High *GATA6* Expression Results in a Reduced Cloning Efficiency (A) Percentage cloning efficiency of each cell fraction (3+/6−, 3+/6L, 3+/6H, and 3−/6+) using OCT4 (left) and SOX2 (right) as markers for the stem cell state. Sorted fractions were plated as single cells at clonogenic density in KO/SR and MEF conditions. Cloning efficiency was calculated by dividing the number of OCT4-positive (left) or SOX2-positive (right) colonies by starting seed density. Error bars represent SD of three biological experiments. Student’s t test was used to determine significance (OCT4 graph: 3+/6− to 3+/6H *p = 0.0017, 3+/6− to 3−/6+ ^∗∗^p = 0.0001, SOX2 graph; 3+/6− to 3−/6H ^∗^p = 0.0019, 3+/6− to 3−/6+ ^∗^p = 0.0002). (B) Proportion of OCT4-positive (OCT4[+]) cells in OCT4-positive colonies derived from single cells from fractions 3+/6−, 3+/6L, 3+/6H, and 3−/6+. Positive colonies include one or more OCT4(+) cells. Counts are shown as bar plots (blue) with superimposed estimated nonparametric distribution (red). (C) Kullback-Leibler symmetric divergence between OCT4-associated distributions shown in (B). This measure increases with reduced similarity between distributions; zero indicates identical distributions. (D) Proportion of SOX2(+) (SOX2-positive) cells in SOX2-positive colonies derived from single cells from fractions 3+/6−, 3+/6L, 3+/6H, and 3−/6+. Positive colonies include one or more SOX2(+) cells. Counts are shown as bar plots (blue) with superimposed estimated nonparametric distribution (red). (E) Kullback-Leibler symmetric divergence between SOX2-associated distributions shown in (D).

Whereas the gene expression patterns of the *GATA6-*expressing subsets suggest progressive endoderm differentiation, the continued expression of the stem cell surface antigen, SSEA3, as well as transcription factors, such as OCT4 and SOX2, is consistent with the retention of an undifferentiated hESC phenotype. To test this, we carried out high-content clonogenic assays to test the self-renewal capacity of single cells from the four cell subsets. Cells from each population (3+/6−, 3+/6L, 3+/6H, and 3−/6+) were isolated by fluorescence activated cell sorting (FACS) and seeded at a clonal density (500 cells/cm^2^) (Blauwkamp et al., 2012). After 4 days, resulting colonies were immunolabeled for expression of OCT4 or SOX2 and the number and characteristics of the colonies were analyzed using a high-content microscopy platform. Colonies were generated from each substate, including the 3−/6+ subset, though with different efficiencies. The cloning efficiencies of the 3+/6− and 3+/6L subsets were similar at around 6%, whereas the cloning efficiency of the 3+/6H cells was lower at about 2.5% and that of the 3−/6+ cells substantially lower at 0.2% (Figure 3A). We also performed the same experiments on the *GATA6* reporter line S4G6 4/F-9 and found the same trend in cloning efficiencies (Figure S3A), demonstrating no effects resulting from the presence of the selection marker. We next looked at the distribution of OCT4 expression within colonies from each of the four cell subsets. For each subset, most cells in each colony expressed OCT4, although there was a noticeable downward shift in the proportion of OCT4(+) cells per colony from the 3+/6− and 3+/6L subsets to the 3+/6H and 3−/6+ subsets, as quantified using the Kullback-Leibler divergence analysis (Figures 3B and 3C). A similar pattern was observed with SOX2 expression, although in all cases there was a broader distribution of SOX2 expression and a significant number of colonies, especially from the 3+/6H and 3−/6+ subsets, contained only SOX2-negative cells, likely due to the absence of SOX2 expression in endoderm differentiation (Adachi et al., 2010) (Figures 3D and 3E). Thus, from these functional studies, we found that the 3+/6H and 3−/6+ subsets had a reduced cloning efficiency, implying a greater tendency to differentiate. Nevertheless, a proportion of cells within these subsets retained the ability to remain within the stem cell compartment and self-renew irrespective of their high *GATA6* expression.

As a more robust assay for confirming that *GATA6*-expressing stem cells were indeed *bona fide* stem cells, we sorted single cells from each subset (3+/6−, 3+/6L, and 3+/6H) into individual wells of a 96-well plate to generate clonal lines. From this, we obtained respectively 43, 76, and 49 clones from 288, 960, and 1,920 cells deposited, equivalent to cloning efficiencies of 15%, 8%, and 3% (Figure S3B). We did not include the 3−/6+ fraction in this part of the study due to its very low cloning efficiency. To check the accuracy of FACS sorting, we used exactly the same conditions to sort mixtures of Chinese hamster ovary (CHO) cells, stably transfected to constitutively express GFP or Tomato fluorescent protein, alongside the sorting for the stem cell fractions (Figure S3C). Using this CHO assay, we detected a misclassification rate of only 1 in every 166 cells sorted (0.6%) (Figure S3D). Based on this rate, as well as the fact that CHO cells have a much higher cloning efficiency than hESCs, thereby over-representing misclassification, we concluded that it was highly unlikely that any clones from the *GATA6*-positive fractions arose from misclassified *GATA6*-negative cells.

**Figure 4:**
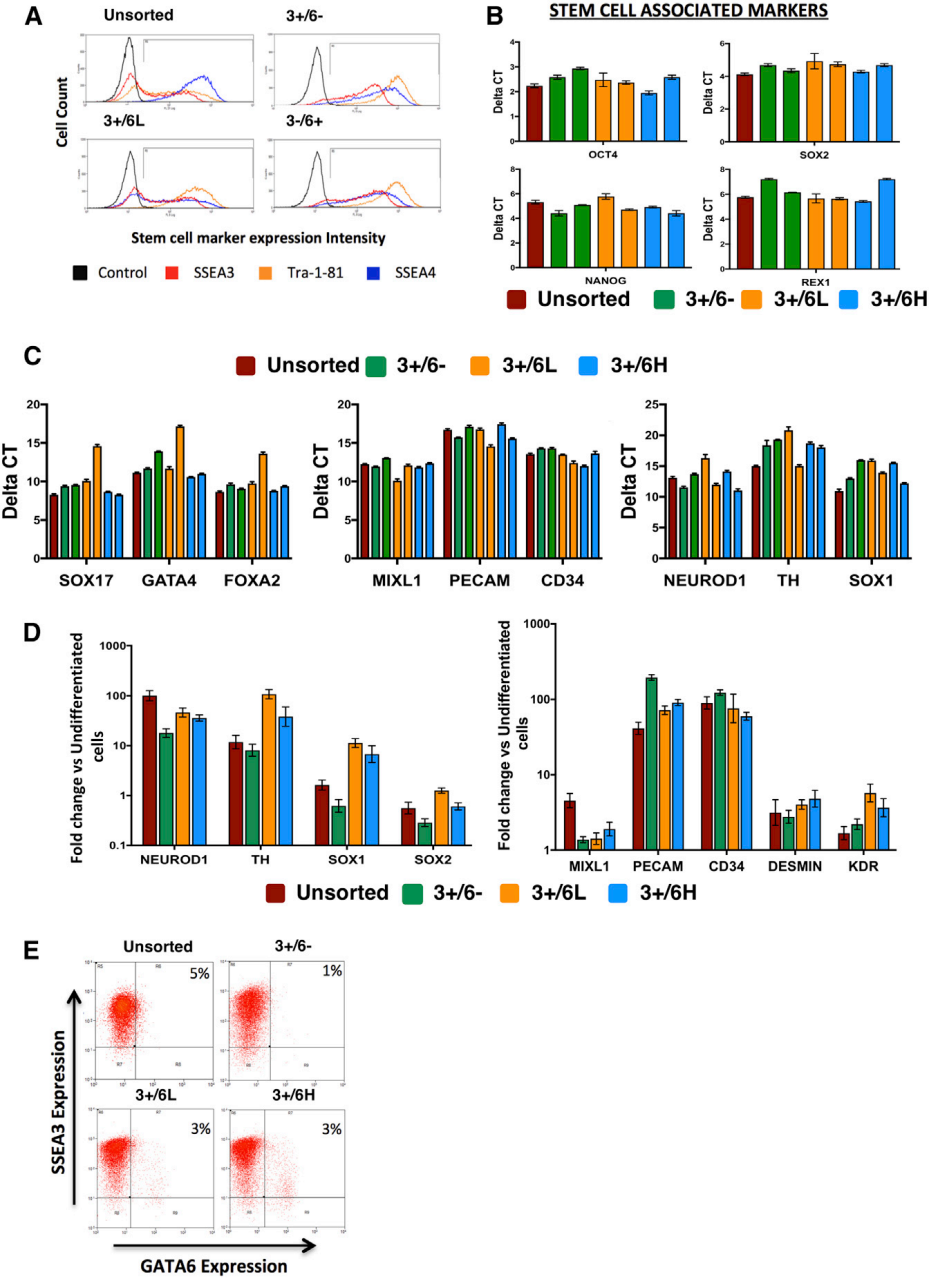
Stable, Long-Term Self-Renewing hESC Subclones Can Be Derived from *GATA6-*Expressing Cells (A) Flow cytometric analysis of subclones derived from the 3+/6−, 3+/6L, and 3+/6H fractions. Unsorted represents the unsorted cells of the reporter line. P3X was used as a negative control, and markers SSEA3 (red), TRA-1-81 (orange), and SSEA4 (blue) were used to identify stem cells. FACS plots show one clone from each fraction, which is representative of four clones analyzed from each fraction. (B) qPCR analysis of two subclones from each fraction for core stem cell transcription factors, shown as Delta-CT normalized to beta-actin; error bars are the SD from three technical repeats. Red bar represents the reporter line, and individual 3+/6−, 3+/6L, and 3+/6H subclones are shown by green, orange, and blue bars, respectively. (C) qPCR for lineage-specific markers of each germ layer in unsorted (red) and two subclones from each fraction. Bar color as in (B), showing Delta-CT normalized to beta-actin with three technical repeats. (D) qPCR of day 10 EBs from unsorted (red), and subclones from 3+/6− (green), 3+/6L (orange), and 3+/6H (blue) fractions for genes specifying mesoderm (left panel) and ectoderm (right panel) to demonstrate pluripotency of the lines. Data shown as fold change against undifferentiated cells from the same starting population. Error bars are SD of three technical repeats. (E) Flow cytometric analysis of reporter line (top left) and 3+/6− (top right), 3+/6L (bottom left), and 3+/6H (bottom right) subclones for SSEA3 versus *GATA6* expression 5–8 passages after initial single-cell seeding. Gates were set using P3X and Shef4 parental line as SSEA3 and GFP negative controls respectively. Plot shows one clone representative of four clones analyzed from each fraction.

All of the clones obtained from each subset grew with a characteristic morphology consistent with that of undifferentiated stem cells (Figure S3E). To confirm this phenotype, six clones were picked from each subset and passaged for a minimum of eight passages with no loss of stem cell morphology. Between passages 5 and 8, two representative clones from each fraction were analyzed by flow cytometry and qPCR for stem cell attributes. Irrespective of the subset of origin, all clones showed similar patterns of SSEA3, TRA-1-81, and SSEA4 expression to that of the unsorted stem cell line (Figure 4A), and expressed similar levels of core stem cell transcription factors OCT4, NANOG, SOX2, and REX1 (Figure 4B). Additionally, gene expression for germ layer differentiation within all subclones was low and comparable to the unsorted line (Figure 4C). To ensure that the clones from each fraction were pluripotent, two representative clones from each were induced to differentiate through a defined, neutral embryoid body differentiation protocol (Ng et al., 2008). Each clone, irrespective of the starting cell, showed strong upregulation of genes associated with mesoderm and ectoderm, demonstrating pluripotency (Figure 4D). Thus, clonal lines generated from hESCs expressing *GATA6* at low and high levels were *bona fide* pluripotent stem cells. Finally, the clones, irrespective of their original *GATA6* status, were able to reconstitute entirely the original culture heterogeneity, so that they were indistinguishable from the starting population after five passages, demonstrating that the *GATA6*-positive substate within the stem cell compartment is interconvertible (Figures 4E and S4).

### *GATA6*-Expressing hESCs Are Biased Toward Endoderm Differentiation

To test whether the hESC subsets expressing *GATA6* exhibit a bias in their propensity to differentiate toward endodermal derivatives, cells from each fraction were isolated by FACS and allowed to differentiate using a defined spin-embryoid body (EB) system without the addition of exogenous proteins or small molecules to direct differentiation. The resulting EBs exhibited structural organization consisting of an inner, middle, and outer mass of cells but there were marked differences in the morphology depending upon the subset of cells from which they were derived. EBs from the 3+/6− and the 3+/6L subsets were similar with a dense, compacted morphology and clear borders. By contrast, the EBs from the 3+/6H and 3−/6+ subsets were much more cystic and showed less structural organization (Figure S5A).

**Figure 5:**
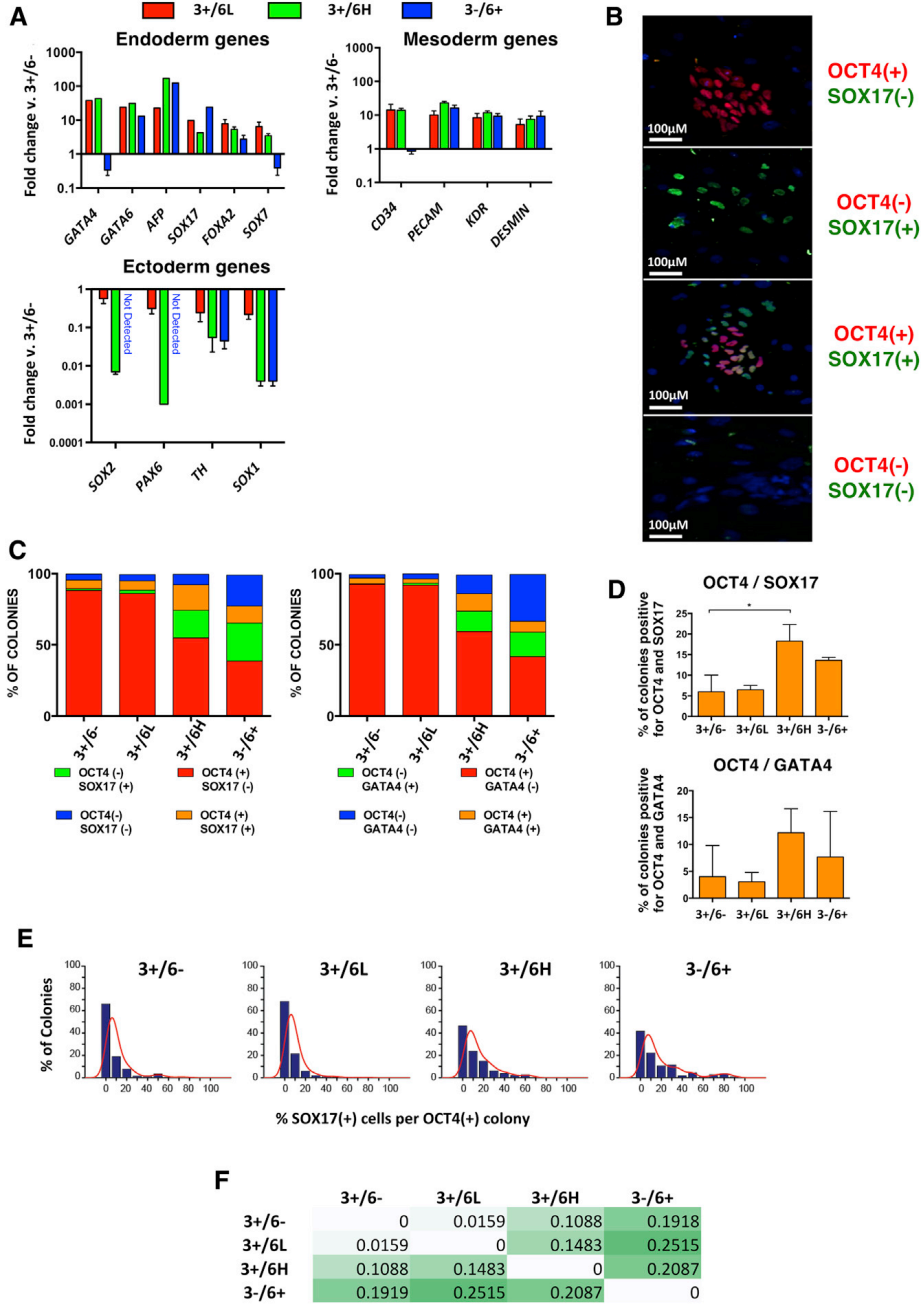
High *GATA6* Expression Results in Endoderm Differentiation Bias at Population and Single-Cell Level (A) qPCR of differentiating cells from the 3+/6L (red), 3+/6H (green), 3-/6+ (blue) fractions in a non-directed EB differentiation assay, shown as fold change against differentiating cells from 3+/6− fraction, for genes expressed in endoderm (top left), mesoderm (top right), and ectoderm (bottom left). Beta-actin was the normalizing gene. Error bars represent three biological replicates. (B) Representative images of colonies derived from 3+/6−, 3+/6L, 3+/6H, and 3−/6+ fractions. Images were taken at ×10 magnification on an InCell Analyzer 2000 and automated quantitative analysis performed using developer toolbox software. The same algorithms were used for each technical and biological repeat and the process was automated to eliminate human bias. (C) Quantification of colony types from 3+/6−, 3+/6L, 3+/6H, and 3−/6+ fractions showing the percentage of colonies per fraction with colony phenotype shown in (B) from three biological repeats. (D) Percentage of colonies containing OCT4 and SOX17 (top graph) or OCT4 and GATA4 (bottom graph) positive cells only. Significance was calculated using t test of three biological replicates and stars represent degree of significance (^∗^p < 0.05). Numbers for each fraction: 3+/6− = 83, 3+/6L = 103, 3+/6H = 122, 3−/6+ = 66. (E) Histogram showing the distribution of SOX17(+) cells in OCT4-positive colonies resulting from single cells from 3+/6−, 3+/6L, 3+/6H, and 3−/6+ fractions. Positive colonies include at least two OCT4(+) cells. Counts are shown as a bar plot (blue) with superimposed estimated nonparametric distribution (red). (F) Kullback-Leibler symmetric divergence between SOX17-associated distributions in OCT4-positive colonies. This measure increases with reduced similarity between distributions; zero indicates identical distributions.

Next, we performed qPCR on day 10 EBs from each subset. Compared with EBs of the 3+/6− subset, EBs from all of the *GATA6* expressing subsets, including 3+/6L cells, showed a marked upregulation of endoderm (*GATA4*, *GATA6*, *AFP*, *SOX17*, *FOXA2*, *SOX7*) and mesoderm-associated genes (*CD4*, *PECAM*, *KDR*, and *DESMIN*). Exceptions were reduced levels of *GATA4*, *SOX7*, and *CD34* in the EBs from the 3−/6+ subsets, potentially due to these cells being further along in differentiation, past the point of normal developmental expression of these genes. By contrast, genes associated with ectodermal differentiation (*SOX2*, *PAX6*, *TH*, and *SOX1*) were markedly downregulated in EBs from the *GATA6-*expressing subsets, with a notable gradation from 3+/6L to 3+/6H and the 3−/6+ derived EBs (Figure 5A).

These results indicate that, on a population basis, the *GATA6* expressing subsets show a strong bias toward endoderm and mesoderm differentiation, at the expense of ectoderm differentiation. Together with the data that these subsets also contain long-term self-renewing undifferentiated stem cells, the results are consistent with the conclusion that, within the stem cell compartment, undifferentiated hESCs can transit reversibly between *GATA6*-positive and *GATA6*-negative substates, but while in these substates they exhibit a differential bias in the pathways of differentiation they are likely to follow. However, the possibility that the *GATA6*-positive subsets contain both undifferentiated, unbiased stem cells together with cells already committed to an endodermal fate, cannot be excluded and may account for the differentiation bias. To address this, we carried out a high-content clonogenic assay to assess the differentiation propensity of individual hESCs under conditions that did permit limited spontaneous differentiation.

Cells from the 3+/6−, 3+/6L, 3+/6H, and 3−/6+ subsets were isolated by FACS and seeded at a clonogenic density of 500 cells/cm^2^ into self-renewing conditions (Barbaric et al., 2014, Blauwkamp et al., 2012). The resulting colonies were dual stained for expression of OCT4, as an indicator of undifferentiated stem cells, and an early endodermal marker, SOX17 or GATA4. Four emerging colony types with respect to SOX17 were apparent, and classified as OCT4(+)/SOX17(−), OCT4(−)/SOX17(+), OCT4(+)/SOX17(+) and OCT4(−)/SOX17(−) (Figure 5B). A similar set with respect to GATA4 expression was also identified (not shown).

OCT4(+)/SOX17(−) colonies or OCT4(+)/GATA4(−) colonies predominated among those derived from 3+/6− and 3+/6L cells compared with fewer such undifferentiated colonies from the 3+/6H and 3−/6+ subsets, as we previously observed. On the other hand, considerably more colonies that contained SOX17 or GATA4 expressing cells were found among those originating from 3+/6H or 3−/6+ cells, consistent with the population differentiation data. Importantly, however, among these fractions was also a higher proportion of SOX17 or GATA4-expressing colonies that also contained OCT4-expressing cells, particularly in the colonies derived from 3+/6H cells (Figures 5C and 5D). We also repeated this experiment on the SG4 4/F-9 reporter clone and found a similar trend (Figure S5B). By looking at the distribution of SOX17 or GATA4 in OCT4-positive colonies from each subset, we also found that there was a small yet distinct increase in the proportion of SOX17(+) or GATA4(+) cells per OCT4-positive colony within the 3+/6H and 3−/6+ biased fractions (Figures 5E, 5F, S5C, and S5D). Taken together, these results indicate that the 3+/6H and even the 3−/6+ subsets contain individual undifferentiated stem cells that exhibit an endoderm differentiation bias.

### Single-Cell Transcriptomic Analysis of Endodermally Biased hESCs

**Figure 6:**
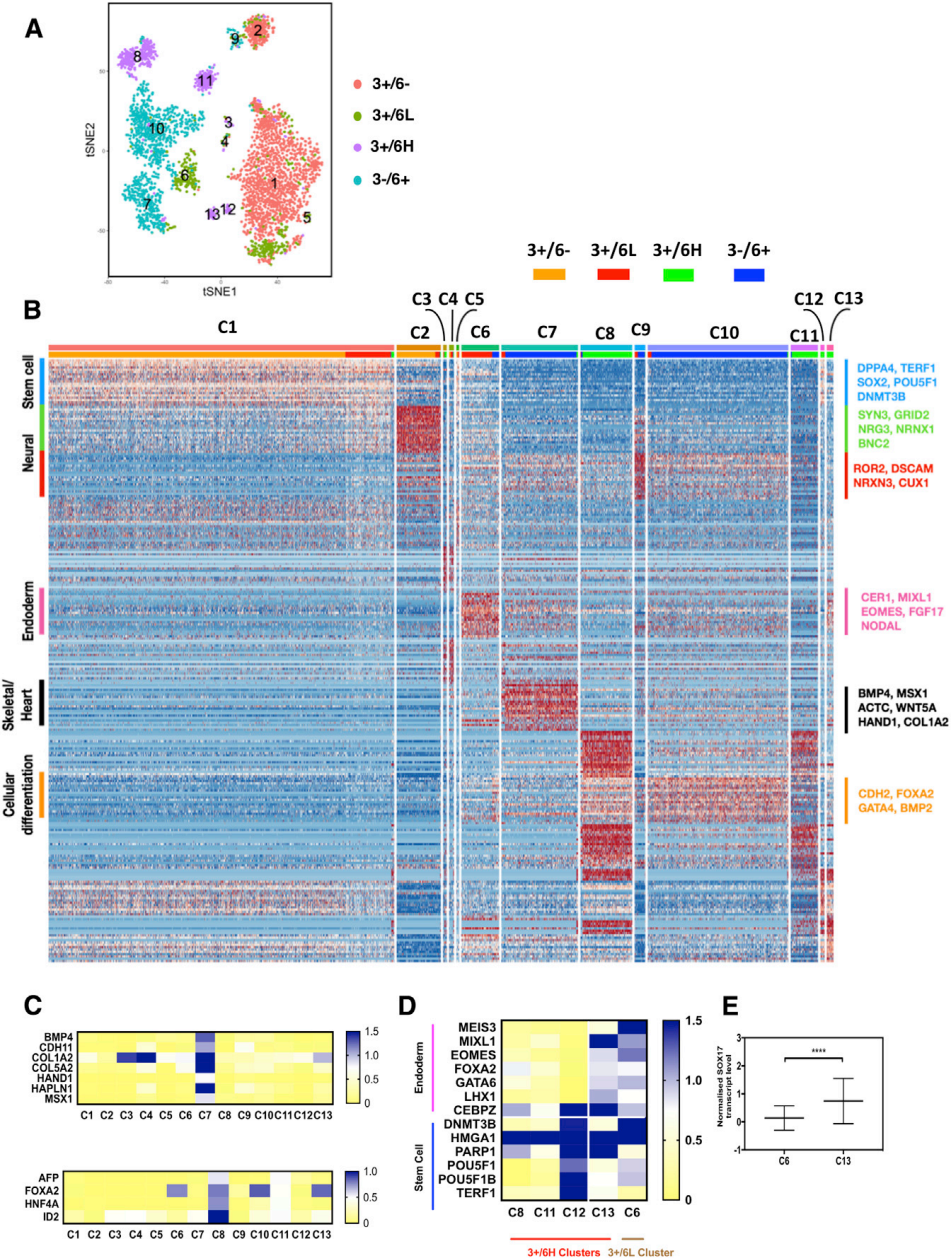
Single-Cell Transcriptomic Analysis of Endodermally Biased hESCs (A) tSNE analysis of the four cellular subsets representing 13 putative clusters separated according to gene expression per single cells. Single cells are represented by individual dots and are colored according to cell fraction library. Numbers represent cluster number assigned arbitrarily. (B) Heatmap of the top 30 most differentially expressed genes between the 13 individual putative clusters, as described in Figure 6A. Color scheme is based on *Z* score distribution from −2 (blue) to +2 (red). Right margin color bars represent gene sets specific to each cluster. Left margin color bars represent top Gene Ontology terms of the top 30 most differentially expressed genes for each cluster. (C) Heatmap of the average expression of genes typically associated with later developmental processes including heart/skeletal (top) and hepatic (lower) lineages across the 13 clusters. Color scheme is based on the averaged normalized expression of each gene from no expression (yellow) to expression (blue). (D) Heatmap of the average expression of genes associated with both the stem cells and early differentiating cells across all the individual 3+/6H clusters and the 3+/6L cluster. Color scheme is based on the averaged normalized expression of each gene from no expression (yellow) to expression (blue). (E) Scatter dot plot to show the mean, upper, and lower limit expression of SOX17 between cluster 6 of the 3+/6L and cluster 13 of the 3+/6H fractions. Student's t test was used to determine statistical significance of ^∗∗∗∗^p > 0.0001.

Having established at the single-cell level that a distinct endoderm-biased substate exists within the stem cell compartment, and with evidence that these four cell fractions represent discrete developmental stages (Figures 2A and 2B), we performed single-cell RNA sequencing, using the Drop-seq methodology (Macosko et al., 2015) on each of the four cell fractions to gain a mechanistic understanding of the populations of cells comprising each fraction. Using tSNE (t-distributed stochastic neighbor embedding) analysis, we defined 13 distinct cell clusters comprising 3,500 cells from all four cell fractions (Figure 6A). We mapped clusters back to cell fraction of origin, and found that clusters were generally fraction specific, so that 3+/6− were confined to clusters 1 and 2, 3+/6L to clusters 1, 5 and 6, 3+/6H to clusters 8, 11, 12, 13, and 3−/6+ to clusters 7, 9, and 10 (Figure 6A). Nevertheless, we saw some overlap of cell fractions within single clusters, particularly for the 3+/6L fraction in clusters 1, 2, 7, 9, and 10, and 3−/6+ in clusters 6 and 8. To ensure these observations were not due to FACS sorting misclassification, we looked at the expression of *GATA6* across the tSNE space and found that *GATA6* was only expressed in clusters composed of GFP(+) sorted cells (Figure S6A). Further, other endoderm-specific genes were only present in GFP(+) sorted cells and strongly correlated with *GATA6* expression (Figure S6B). Thus, the single-cell data showed further heterogeneity within sorted cell fractions as evidenced by the generation of multiple clusters per fraction, and it was apparent that some cells within a fraction showed more transcriptomic similarities to cells of other fractions.

As an unbiased approach to investigate which cell types were being generated in the cell fractions, we performed cluster-specific binomial differential gene expression analysis. We found that the 3+/6− fraction in cluster 1 showed the highest level of stem cell-associated gene expression. Interestingly, we found that cluster 2, although derived from the 3+/6− fraction, showed strong upregulation of neural associated genes including *SYN3*, *GRID2,* and *NRG3*. Cells of the 3+/6L fraction, which showed similar self-renewal behavior to cells of 3+/6− were split between cluster 1 and cluster 6, whereby both clusters showed high stem cell gene expression. The 3+/6L cells within cluster 6, however, also expressed high levels of early endoderm-associated genes, including *EOMES*, *FGF17*, *NODAL*, and *LEFTY1*, which may account for the observed endoderm differentiation bias within our neutral EB differentiation assay. The 3+/6H fraction, except clusters 12 and 13, and the 3−/6+ fractions consisted of cells with low stem cell expression yet high expression of genes involved in cellular differentiation, gastrulation, and endoderm (clusters 7–11), consistent with their general lack of ability for self-renewal (Figure 6B). Additionally, it was apparent that *GATA6* expression correlated with multiple lineages, including mesoderm (cluster 7), and definitive endoderm (clusters 8 and 11) differentiation, although we found no strong evidence for primitive endoderm by *SOX7* expression (Figure S6C). Further, cells within the 3+/6H and 3−/6+ fractions generated clusters that showed higher expression of more mature endoderm-associated genes (*AFP*, *FOXA2*, *ID2*, and *HNF4A*; cluster 8) and mesoderm-associated genes (*MSX1*, *HAND1*, *CDH11*, and *ALPK2*; cluster 7) (Figure 6C), confirming our previous observations that these cell fractions represent a later developmental time point than the 3+/6L and 3+/6− fractions. Thus, these data enabled us to capture discrete subpopulations of cells progressing along a developmental trajectory that correlates with the increased expression of *GATA6* and the subsequent loss of SSEA3. We next sought to identify which cluster may represent the endoderm-biased stem cells of the 3+/6H fraction. Of all the clusters composed of 3+/6H cells, only cluster 13 had robust and significant co-expression of both endoderm and stem cell genes (Figure 6D). Further, cells within cluster 13 also showed co-expression of *OCT4*, *SOX2*, and *GATA6*, indicative of mesendodermally biased cells (Nazareth et al., 2013) (Figure S6E). This cluster, however, was not unique in the sense that cluster 6 of the 3+/6L fraction also showed strong co-expression of genes for these opposing lineages, but did not show functional bias toward endoderm differentiation in our single-cell assay. To investigate what specific genes may be driving this unique biased state of the 3+/6H cells at single-cell level, we performed pairwise differential expression analysis between clusters 6 and 13, and then filtered results for transcription factors. We identified one transcription factor gene, *SOX17*, as significantly more highly expressed in the 3+/6H fraction compared with the 3+/6L (Figure 6E). Therefore, it appears that the retention of expression of stem cell genes is imperative to remain within the stem cell compartment, and *SOX17* may be a main driving force for cells to enter an endoderm-biased substate.

## Discussion

Using a *GATA6*-GFP reporter line, we have corroborated our previous observations (Gokhale et al., 2015) and confirmed in live cells that *GATA6* is heterogeneously expressed in a subset of cells alongside the surface stem cell marker SSEA3. GATA6 is a key lineage-associated transcription factor implicated in specifying the endoderm lineage during the segregation of the inner cell mass and extra-embryonic lineages in the blastocyst; later during gastrulation, it is expressed in cells of the lateral plate mesoderm (Koutsourakis et al., 1999). On the other hand, SSEA3 is associated with a cell surface globoseries glycolipid expressed by undifferentiated hESCs (Andrews et al., 1982, Kannagi et al., 1983). Compared with other surface markers of these cells, SSEA3 is lost most quickly upon differentiation (Draper et al., 2002, Enver et al., 2005, Fenderson et al., 1987).

Our results demonstrate that undifferentiated hESCs can transiently express a lineage regulatory transcription factor, GATA6, while retaining the capacity for long-term self-renewal. Further, these undifferentiated stem cells can oscillate between a *GATA6*-positive and *GATA6*-negative expression state. Also, on a population basis, when differentiation was induced by EB formation, the *GATA6*-positive cells showed a greater propensity to differentiate toward endoderm-related lineages, than do the *GATA6*-negative cells, which appear to exhibit a greater propensity for ectodermal differentiation. Further, qPCR analysis of these subsets demonstrated that the increased expression of *GATA6* correlated with the increased expression of genes involved in early gastrulation and differentiation. More specifically, genes associated with endoderm but not mesoderm or ectoderm were upregulated, suggesting directional activation of an endodermal program. This pattern of gene upregulation is consistent with the role of GATA6 in the early specification of extra-embryonic endoderm and definitive endoderm during mouse gastrulation (Chazaud et al., 2006, Koutsourakis et al., 1999, Plusa et al., 2008), as well as the expression of *GATA6* in hESC-derived definitive endoderm (McLean et al., 2007). The subsets revealed a clear hierarchy of cells in culture such that the 3+/6− and 3−/6+ fractions showed quite opposite gene expression patterns, with the 3+/6− subset representing a more pristine stem cell state and the 3−/6+, a more differentiated state, with the 3+/6L and 3+/6H subsets in between. The cloning efficiency of these subsets similarly reduced progressively from the 3+/6− subset through the 3+/6L and 3+/6H subsets and was lowest in the 3−/6+ subset implying a corresponding reduction in the proportions of clonogenic stem cells in each subset.

The reduced cloning efficiency and increased propensity for endoderm differentiation of the *GATA6*-positive subsets could be explained by a lineage bias in self-renewing stem cells that co-express pluripotent associated and lineage-associated genes, with a corresponding reduction in cloning efficiency, or it could reflect the presence of two further subsets within each of the 3+/6L and 3+/6H subsets, one self-renewing but not lineage biased and one not self-renewing but committed progenitor cells, as reported by Pina et al. (2012) for hematopoietic stem cells. These possibilities are not mutually exclusive. Unfortunately, given the low plating efficiency of hESCs, it is not possible to conclude directly from population-level data whether this population bias reflects a differentiation bias at the level of individual self-renewing stem cells.

However, using OCT4 or SOX2 as surrogate markers of self-renewing stem cells, in addition to SSEA3, we were able to show that single-cell-derived colonies that we classified as arising from self-renewing stem cells contained more spontaneously differentiated cells of the endoderm pathway, marked either by SOX17 or GATA4, when derived from the 3+/6L or 3+/6H subsets, than when derived from the 3+/6− subset. Further, many of the cells within each colony expressed these stem cell markers implying continued expression through at least four to five cell divisions. We conclude that the colonies classified as OCT4-positive or SOX2-positive were derived from self-renewing undifferentiated embryonic stem cells, and that not only can self-renewing stem cells express the lineage regulator transcription factor GATA6 but also that its expression does increase the probability of those stem cells following an endoderm route when they commit to differentiation.

By single-cell RNA sequencing we are able to identify single cells co-expressing both stem cell- and endoderm-specific genes, beyond that of SSEA3 and *GATA6* alone. Using tSNE analysis, we found that almost all 3+/6L and a small proportion of 3+/6H cells retained the expression of key stem cell-associated genes, likely representing cells within the stem cell compartment and in line with our functional data. In particular, we found that the co-expression of *OCT4* and *SOX2* was strongly retained within self-renewing associated clusters but lost in all other clusters, implicating an important role for OCT4 and SOX2 in the ability for endoderm gene expressing cells to remain within the stem cell compartment. This is supported by the established role of OCT4 and SOX2 as master regulators of the stem cell state (Buitrago and Roop, 2007, Huangfu et al., 2008, Takahashi et al., 2007). Thus, the status of OCT4/SOX2 expression may dictate cellular residence inside or outside of the stem cell compartment. We also found that the 3+/6− fraction showed heterogeneity, consistent with previous functional reports (Tonge et al., 2011). Interestingly, we found a subset of cells, approximately 11% of the 3+/6− fraction, with neural gene expression profiles. We also found co-expression of stem cell genes alongside mesodermal-associated genes, so one could imagine a system that contains pluripotent stem cells biased toward each primary germ layer. To support this hypothesis, however, further work is required to elucidate whether these cells also identify functional lineage-biased substates within the stem cell compartment.

Hierarchies of human pluripotent stem cells based on the co-expression levels of the surface stem cell markers GCTM-2 and CD9 have also shown the existence of lineage marker expression in stem cell populations, albeit with little functional relevance (Hough et al., 2009, Hough et al., 2014). It was suggested that cultures of these cells contain metastable self-renewing cells in a continuum with intermediate pluripotent states that eventually become primed for lineage specification. Our results are similarly consistent with a continuum in which the self-renewing capacity of the stem cells diminishes as they progressively acquire lineage-associated features while retaining the ability to revert to a more pristine, less lineage-associated, state. Evidently, heterogeneity has functional relevance to the behavior of hESCs. With substantial evidence for functional substates within the stem cell compartment, a deeper understanding of the mechanisms that govern and stabilize these substates would offer a new level of control for the efficient and uniform differentiation of hESCs, and so facilitate the development of applications such as in regenerative medicine.

## Experimental Procedures

### Cell Culture

The Shef4 hESC line (Aflatoonian et al., 2010) and its derivatives were cultured on mitomycin C inactivated mouse embryonic fibroblasts in Knockout DMEM with 20% Knockout serum replacement as previously described (Draper et al., 2002) or in feeder-free conditions using E8 medium and vitronectin (Life Technologies). Embryoid bodies were produced and grown in the serum-free, defined medium, APEL (Stem Cell Technologies), as described by Ng et al. (2008). See Supplemental Experimental Procedures for more details.

### Generation of *GATA6*-GFP Reporter hESCs

*GATA6* reporter Shef4 hESCs were generated using a standard gene targeting replacement vector designed to insert an GFP reporter cassette by homologous recombination into exon 2 of the human *GATA6* locus at the position of the ATG translational initiation codon. See Supplemental Experimental Procedures for more details.

### Immunoassays, Flow Cytometry, and Cell Sorting

For details including a list of antibodies, see Supplemental Experimental Procedures.

### Gene Expression Analysis

Quantitative real-time PCR was performed on the QuantStudio 12K Flex Real-Time PCR system (Invitrogen) using TaqMan universal master mix (Invitrogen) in conjunction with the Roche universal probe library system (Roche). Drop-seq analysis was carried out as described in Macosko et al. (2015). For full details including a list of qPCR primers, and analytical methods, see Supplemental Experimental Procedures.

### Statistical Analysis

For full details of statistical tests including clustering, boxplot, Kullback-Leibler divergence analysis, and tSNE analysis of single-cell RNA-sequencing data, see Supplemental Experimental Procedures.

## Author Contributions

T.A. devised experimental plans and performed the bulk of the experiments and interpretation of the results within this manuscript. A.J.H.S., J.S.S., and K.A. generated the targeting construct and subsequently the *GATA6* reporter line. V.B. and S.S. provided the bioinformatics analyses. J.L. provided detailed instructions to establish the single-cell drop-seq technique. D.S. and J.H. performed qPCRs to satisfy reviewer comments. M.J. performed FACS sorting and analysis. K.P., D.C., I.B., P.G., and P.W.A. are principal investigators who helped devise and interpret all experiments and results.

## Accession Numbers

The accession number for the RNA-sequencing data reported in this paper is GEO: GSE113168.

## Supplemental Information

Document S1. Supplemental Experimental Procedures and Figures S1–S6

Document S2. Article plus Supplemental Information
