# Supplementary material for: Identification and Single-Cell Functional Characterization of an Endodermally Biased Pluripotent Substate in Human Embryonic Stem Cells
Source: Stem Cell Reports. 2018 May 17;10(6):1895–907. doi: 10.1016/j.stemcr.2018.04.015 (PMC5993559; doi:10.1016/j.stemcr.2018.04.015)
Supplement: Document S1. Supplemental Experimental Procedures and Figures S1–S6 [file mmc1.pdf]

Stem Cell Reports, Volume *10*

## Supplemental Information

# Identification and Single-Cell Functional Characterization of an Endodermally Biased Pluripotent Substate in Human Embryonic Stem Cells

**Thomas F. Allison, Andrew J.H. Smith, Konstantinos Anastassiadis, Jackie Sloane-Stanley, Veronica Biga, Dylan Stavish, James Hackland, Shan Sabri, Justin Langerman, Mark Jones, Kathrin Plath, Daniel Coca, Ivana Barbaric, Paul Gokhale, and Peter W. Andrews**

# Identification and single cell functional characterization of an endodermally-biased pluripotent sub-state in human embryonic stem cells

Supplemental Figures

## Figure S1

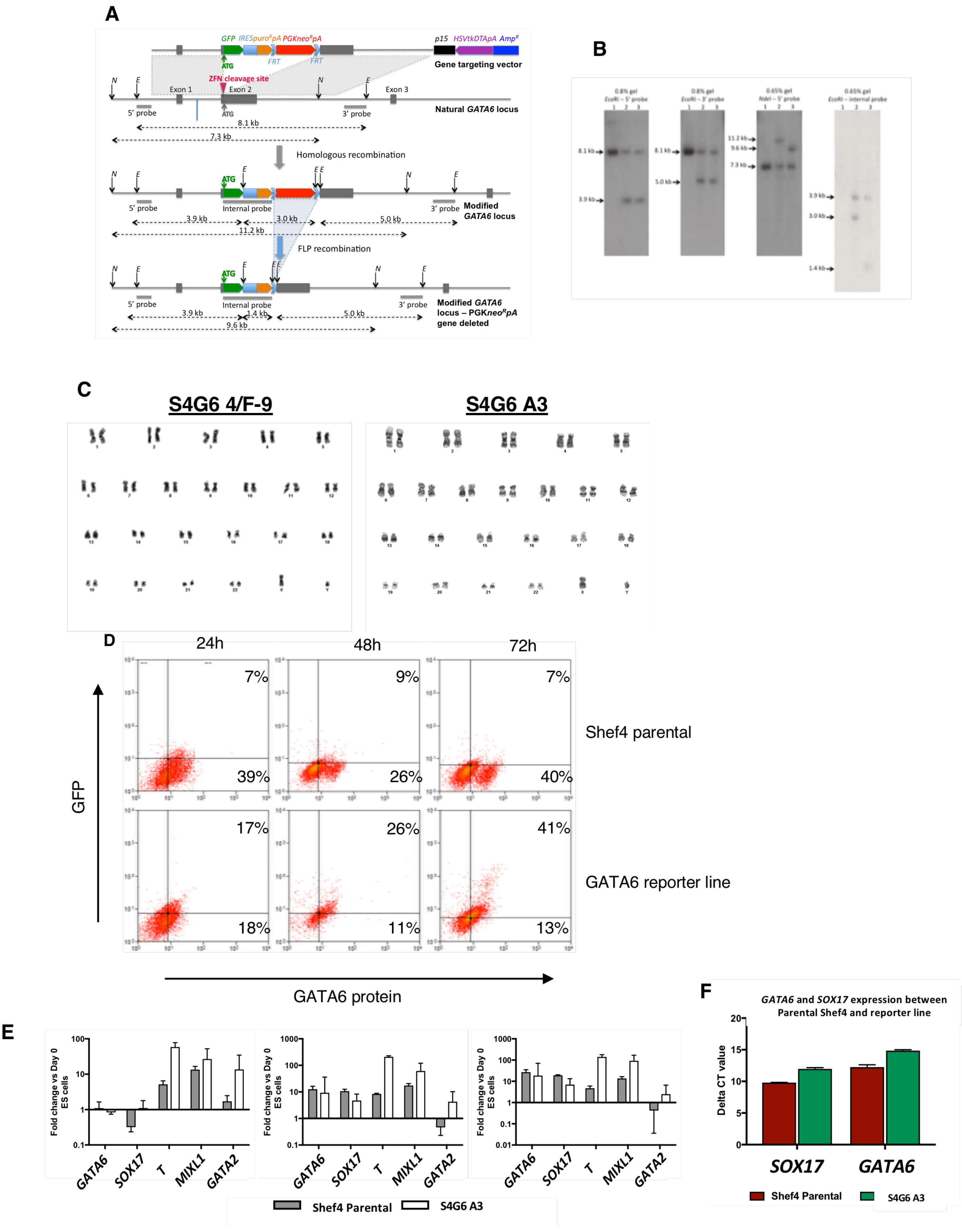

A
GFP
IRESpuroRpA
PGKneoRpA
p15
HSVtkDTApA
AmpR
FRT
ATG
Gene targeting vector
ZFN cleavage site
Exon 1
Exon 2
Exon 3
5' probe
3' probe
Natural GATA6 locus
8.1 kb
7.3 kb
Homologous recombination
Internal probe
Modified GATA6 locus
3.9 kb
3.0 kb
5.0 kb
11.2 kb
FLP recombination
Modified GATA6 locus – PGKneoRpA gene deleted
1.4 kb
9.6 kb
B
0.8% gel
EcoRI – 5' probe
0.8% gel
EcoRI – 3' probe
0.65% gel
NdeI – 5' probe
0.65% gel
EcoRI – internal probe
C
S4G6 4/F-9
S4G6 A3
D
24h
48h
72h
7%
39%
9%
26%
7%
40%
Shef4 parental
17%
18%
26%
11%
41%
13%
GATA6 reporter line
GFP
GATA6 protein
E
Fold change vs Day 0 ES cells
GATA6
SOX17
T
MIXL1
GATA2
Shef4 Parental
S4G6 A3
F
GATA6 and SOX17 expression between Parental Shef4 and reporter line
Delta CT value
SOX17
GATA6
Shef4 Parental
S4G6 A3

**Figure S1**

**(A)** Diagram of the linearized gene targeting vector, and the stages of genetic modification of the *GATA6* locus. Homology regions between the gene targeting vector and the *GATA6* locus within which homologous recombination can occur are indicated by the grey shaded regions. The *GATA6* gene targeting vector is designed for positive-negative selection with an internal constitutively expressed neomycin resistance-positive selection cassette (*PGKneo$^R$pA)* and, on the terminus of the right homology arm, a constitutively expressed Diphtheria Toxin A (DTA) chain negative selection cassette (*HSVtkDTApA*). The vector contains the enhanced Green Fluorescent Protein (GFP) reporter gene inserted at the position of the ATG translational initiation codon in the sequence of *GATA6* exon 2. Integration by homologous recombination is designed to result in expression of the enhanced GFP reporter and the puromycin resistance (*puro*$^R$*pA*) gene (linked via an Internal Ribosome Entry Site [IRES] sequence) under transcriptional control from the endogenous *GATA6* locus. The polyadenylation addition signal sequence (pA) at the 3' end of the puromycin resistance gene is predicted to result in premature termination of the *GATA6* transcript from the gene targeted allele. Subsequently the *PGKneo$^R$pA* selection cassette was deleted via the flanking *FRT* recombination sites by FLP site-specific recombination (indicated by the grey shaded triangle). Positions of *Eco*RI (*E*) and *Nde*I (*N*) restriction enzyme sites are shown by vertical arrows with predicted restriction fragments shown by horizontal dashed lines with arrowheads and sizes in kilobases (kb). Positions of the flanking 5' and 3' probes, and the internal probe, used for Southern blot analysis are shown as grey bars. **(B)** Southern blot analysis to confirm correct genetic modification of the *GATA6* locus. In all Southern blots: genomic DNA from the starting Shef4 cells before transfection - track 1; heterozygous *GATA6* gene targeted clone S4G6 4/F-9 with the *PGKneoRpA* selection marker present - track 2; derivative heterozygous *GATA6* gene targeted clone S4G6 A3 with the *PGKneoRpA* selection marker deleted by FLP recombination - track 3. Genomic DNAs were cut with *Eco*RI and *Nde*I and fractionated on 0.8% and 0.65% gels as indicated. Filters were hybridized with 5' and 3' flanking probes, and with the internal vector probe, as indicated. Sizes of bands were determined using a λ *Hind*III marker track (not shown). Restriction fragment sizes are consistent with predicted sizes shown in Supplementary Figure 1A. The internal probe only detects the predicted restriction fragments and importantly no additional restriction fragment sizes are detected proving that there is no random integration of the vector and the only reporter insertion is at the *GATA6* locus. **(C)** karyotype analysis of the hESC S4G6 4/F-9 reporter line and the derivative S4G6 A3 reporter line used within this study, demonstrating in both a normal 46XY karyotype. 30 metaphases were scored. **(D)** Time-course differentiation for the parental Shef4 and *GATA6* reporter (S4G6 A3) hESC lines. The parental and *GATA6* reporter Shef4 hESC lines were differentiated using an endodermal differentiation protocol (D'Amour et al., 2005) and FACS analyzed for GFP (Y axis) vs GATA6 protein (X axis) every 24 hours over a 72 hour period. **(E)** qPCR analysis for early primitive streak markers on a time-course differentiation for the parental Shef4 (grey bars) and the Shef4 S4G6 A3 (unfilled clear bars) hESC lines. Fold changes were calculated against respective hESC pluripotent lines, and β-actin was used as a normalizing gene. **(F)** Comparison of *GATA6* and *SOX17* expression levels between the parental Shef4 and S4G6 A3 reporter hESC lines.

## Figure S2

**A**

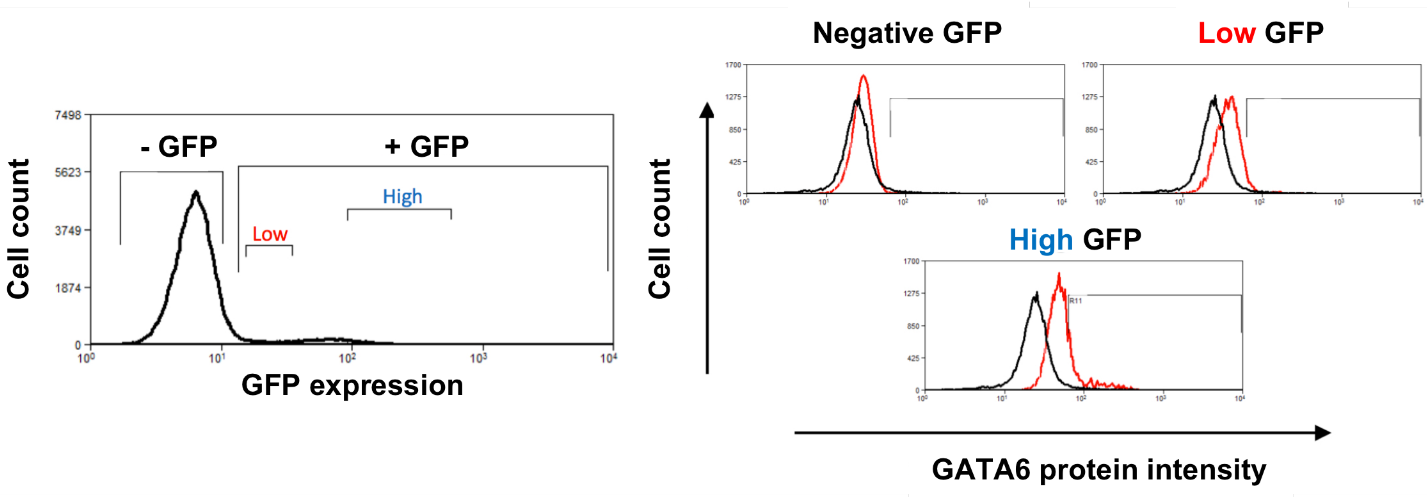


| Gated sample | % of total population | % of cells expressing GATA6 protein |
|---|---|---|
| -GFP | 91% | 0.9% |
| Low GFP | 2% | 5.5% |
| High GFP | 1.5% | 23.2% |

**B**

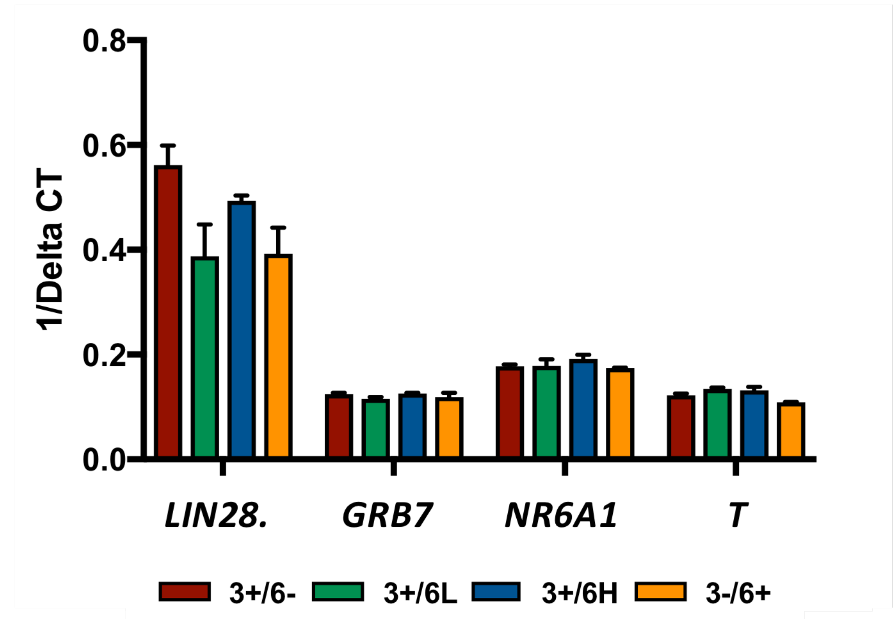


**Figure S2**

**(A)** The left panel shows a representative FACS plot of GFP (*GATA6* mRNA) expression within standard MEF/KOSR stem cell cultures, demonstrating the existence of low and high *GATA6* expressing cells. The right panel shows corresponding immunostaining for GATA6 protein levels within these subsets of low or high GFP (*GATA6* mRNA) expressing cells, demonstrating increased levels of GATA6 protein as GFP (*GATA6* mRNA) increases. Black lines show the isotype control, and red lines show GATA6 protein expression. **(B)** qPCR expression levels of genes that did not show significant changes between the four cell fractions (3+/6-, 3+/6L, 3+/6H and 3-/6+). Data is shown as 1/Delta CT values using β-actin as the normalizing gene, error bars representative of three technical repeats.

**Figure S3**

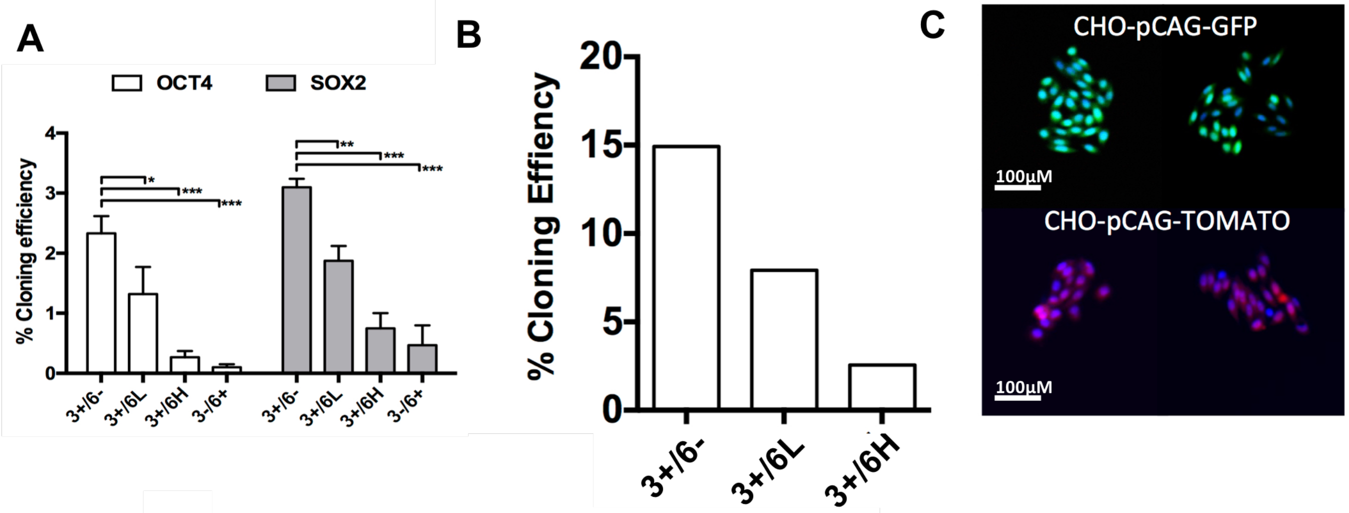


**D**

| Fraction | Number of cells sorted | Possible error clones at 0.6% | Number of clones observed |
|---|---|---|---|
| 3+/6- | 288 | 2 | 43 |
| 3+/6L | 960 | 6 | 76 |
| 3+/6H | 1920 | 12 | 49 |

**E**

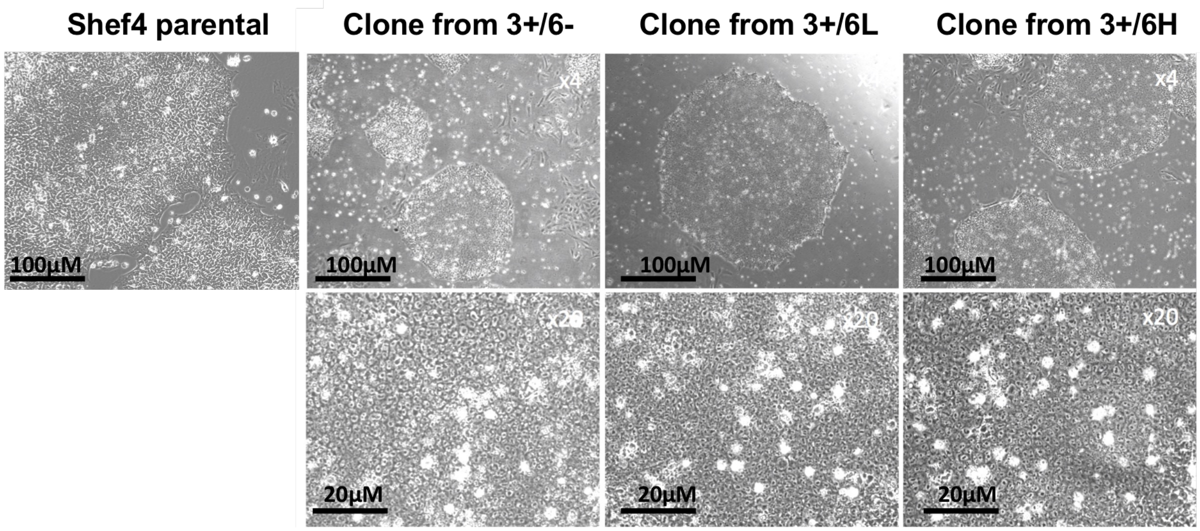


__Figure S3__

**(A)** Cloning efficiency of the sorted 3+/6-, 3+/6L, 3+/6H and 3-/6+ cell fractions using the reporter cell line S4G6 4/F-9. White bars represent colonies stained for OCT4, and grey bars, SOX2. The t-test was used to assess significance and is representative of three biological repeats, with p values of 0.0017 for 3+/6- to 3+/6L, 0.0001 for 3+/6- to 3+/6H and 0.0002 for 3+/6- to 3-/6+.**(B)** Cloning efficiency of the 3+/6-, 3+/6L and 3+/6H cell fractions after single cell/well FACS sorting. **(C)** Representative images of CHO-GFP (left) or CHO-Tomato (right) expressing colonies after single cell deposition used to determine FACS sorting misclassification rates. Single CHO cells were maintained for 4 days to form colonies then fixed to determine the proportion of misclassification, quantified in **(D).** **(E)** Representative images of resulting clones from single cell/well deposition showing typical hESC morphology. Shef4 parental line is shown as the control.

Figure S4

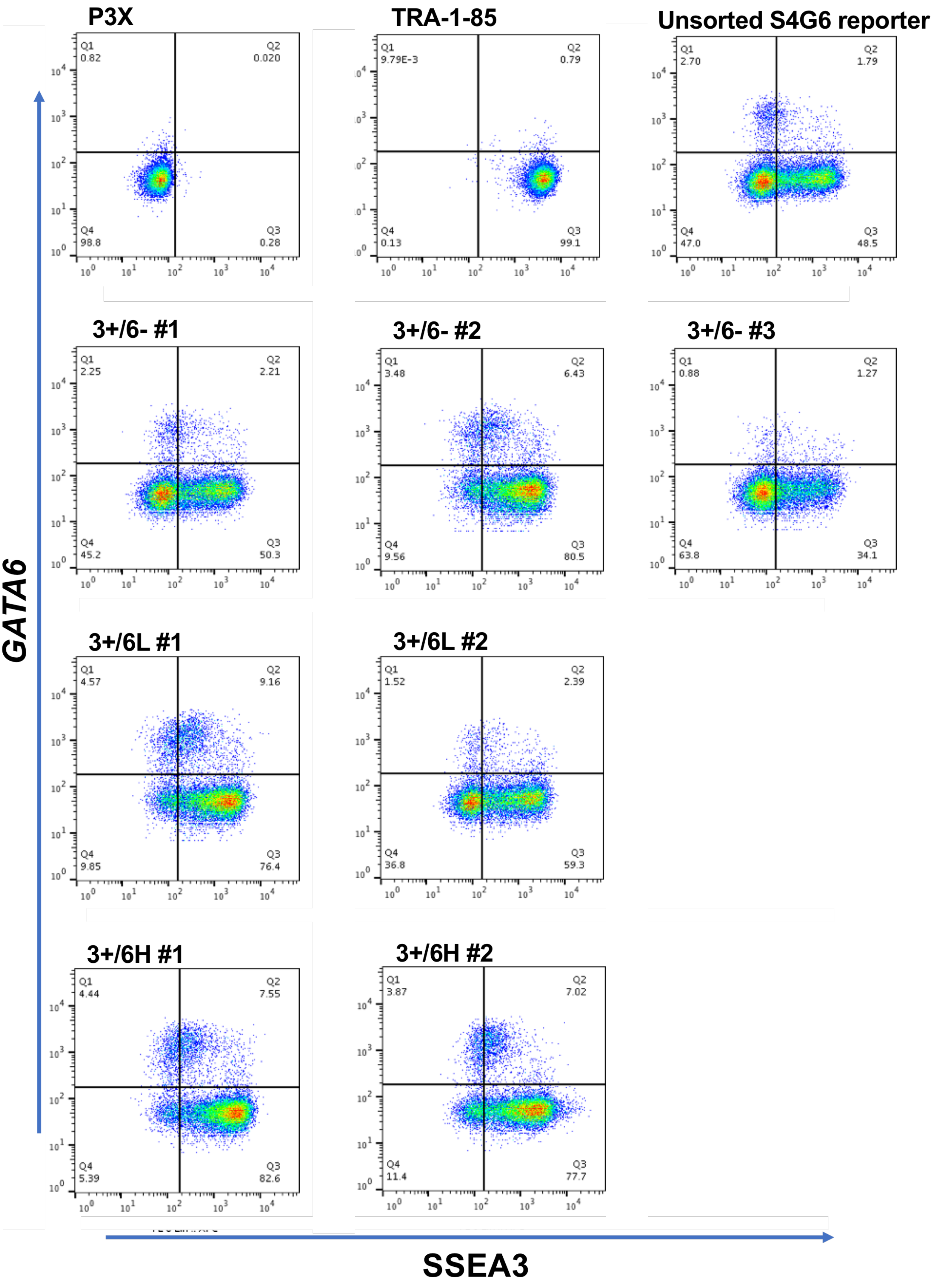

P3X
Q1 0.82
Q2 0.020
Q4 98.8
Q3 0.28
TRA-1-85
Q1 9.79E-3
Q2 0.79
Q4 0.13
Q3 99.1
Unsorted S4G6 reporter
Q1 2.70
Q2 1.79
Q4 47.0
Q3 48.5
3+/6- #1
Q1 2.25
Q2 2.21
Q4 45.2
Q3 50.3
3+/6- #2
Q1 3.48
Q2 6.43
Q4 9.56
Q3 80.5
3+/6- #3
Q1 0.88
Q2 1.27
Q4 63.8
Q3 34.1
3+/6L #1
Q1 4.57
Q2 9.16
Q4 9.85
Q3 76.4
3+/6L #2
Q1 1.52
Q2 2.39
Q4 36.8
Q3 59.3
3+/6H #1
Q1 4.44
Q2 7.55
Q4 5.39
Q3 82.6
3+/6H #2
Q1 3.87
Q2 7.02
Q4 11.4
Q3 77.7
GATA6
SSEA3

**Figure S4**

FACS analysis of clones from the 3+/6-, 3+/6L and 3+/6H cell fractions (two clones from each) derived from the single cell deposition experiment, after five passages in self- renewing conditions, showing the expression of SSEA3 (x-axis) vs *GATA6* (y-axis). P3-X serves as the negative, and TRA-1-85 as positive controls for appropriate FACS gating. All long-term clones show the re-distribution of all cell fractions after prolonged passaging.

Figure S5

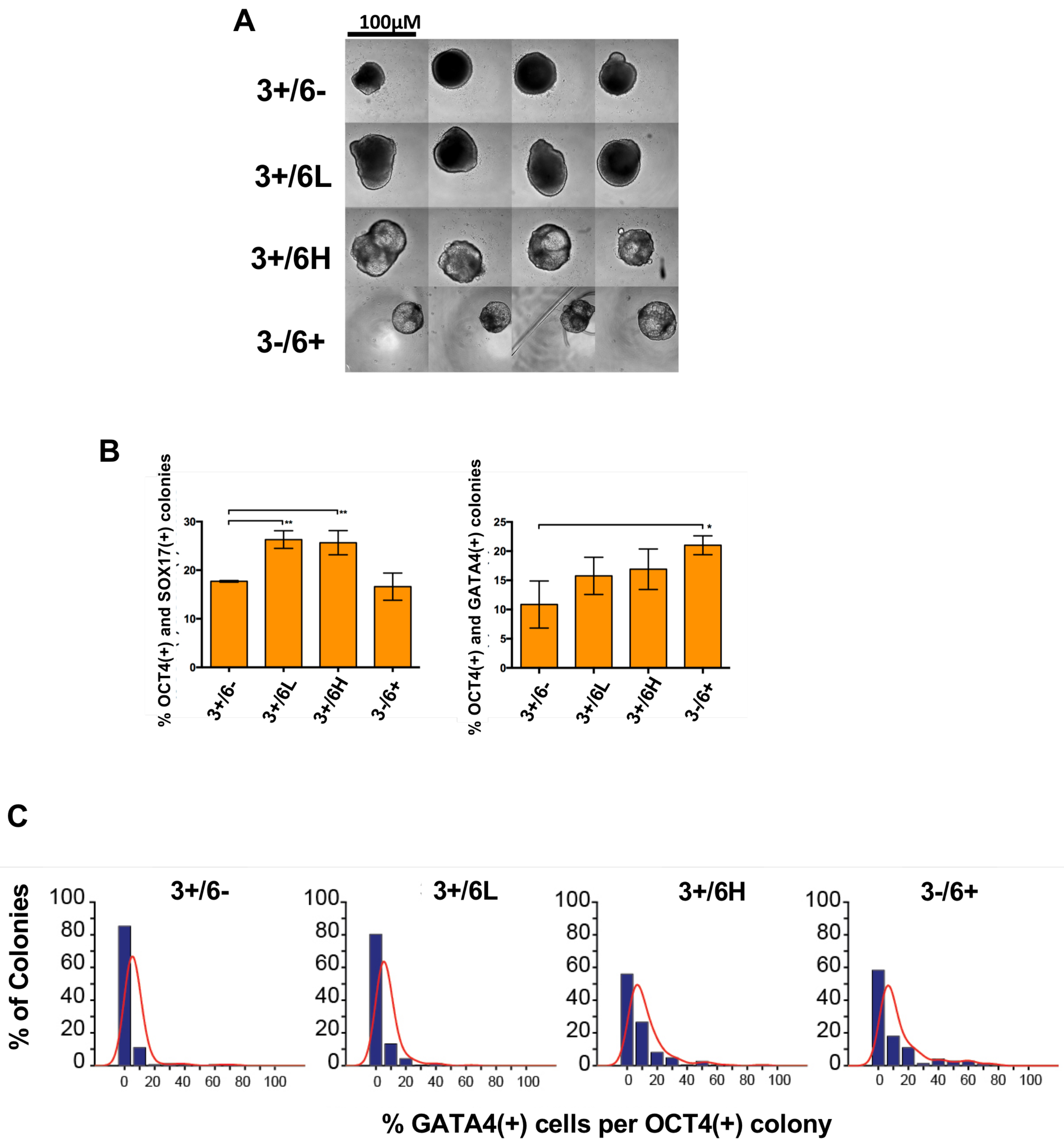


D

| | 3+/6- | 3+/6L | 3+/6H | 3-/6+ |
|---|---|---|---|---|
| 3+/6- | 0 | 0.0251 | 0.2253 | 0.2411 |
| 3+/6L | 0.0251 | 0 | 0.1613 | 0.194 |
| 3+/6H | 0.0225 | 0.1613 | 0 | 0.043 |
| 3-/6+ | 0.2411 | 0.194 | 0.043 | 0 |

**Figure S5**

**(A)** Representative images to demonstrate the differing morphology of embryoid bodies generated from cells from the 3+/6-, 3+/6L, 3+/6H and 3-/6+ cell fractions after 10 days of differentiation. Images were taken at x4 magnification on the InCell Analyzer 2000. **(B)** Percentage of colonies containing both OCT4(+) and SOX17(+) cells (left graph, 3+/6- to 3+/6L p=0.0012, 3+/6- to 3+/6H p=0.0052) or both OCT4(+) and GATA4(+) cells (right graph, 3+/6- to 3-/6+ p=0.015) from the reporter line S4G6 4/F-9. Significance was calculated using t-test of three biological replicates. **(C)** Histogram showing the distribution of GATA4(+) cells in OCT4-positive colonies resulting from single cells from 3+/6-, 3+/6L, 3+/6H and 3-/6+ fractions. Positive colonies include at least two OCT4(+) cells. Counts are shown as a bar plot (blue) with superimposed estimated nonparametric distribution (red) and are representative of three biological replicates. **(D)** Kullback-Leibler symmetric divergence between GATA4-associated distributions in OCT4-positive colonies. This measure increases with reduced similarity between distributions; zero indicates identical distributions.

**Figure S6**

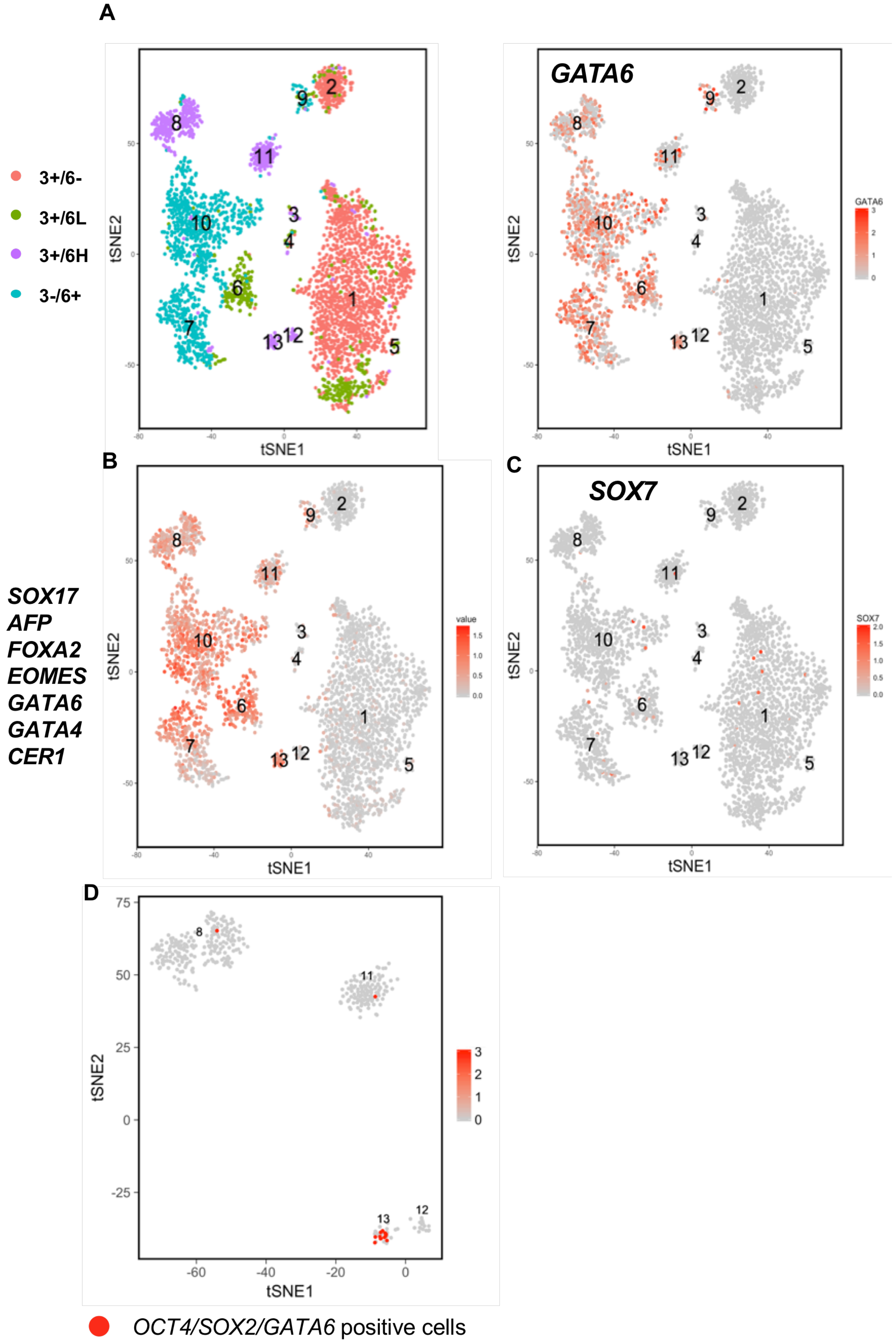
A
3+/6-
3+/6L
3+/6H
3-/6+
tSNE2
tSNE1
GATA6
B
SOX17
AFP
FOXA2
EOMES
GATA6
GATA4
CER1
value
C
SOX7
D
OCT4/SOX2/GATA6 positive cells

**Figure S6**

**(A)** The left panel shows a tSNE plot of single cell data from the four cell fractions, colored according to their original fraction. Each dot represents a single cell and putative clusters are numbered at random. The right panel shows the same tSNE plot overlaid with the expression of *GATA6*, showing only cells from the GFP positive sorted fractions had *GATA6* expression. Red represents *GATA6* positive, and grey, *GATA6* negative cells. **(B)** tSNE plot of all single cells to show the average expression of multiple endoderm associated genes indicating that their expression correlates with *GATA6*. Only cells from the GFP positive cell fractions showed expression of these genes. **(C)** tSNE plot of all single cells showing the expression of the primitive endoderm marker *SOX7*. Red represents *SOX7* positive, and grey, *SOX7* negative cells. **(D)** Reduced tSNE plot showing only the clusters composed of cells from the 3+/6H fraction. Red represents cells that are positive for all of the genes *OCT4*, *SOX2*, and *GATA6*, and grey are cells that do not co-express all three of these genes.

# Supplemental Experimental Procedures

## Cell Culture

Human embryonic stem cells (hESC) were routinely cultured as previously described (Draper et al., 2002) on 0.1% gelatin coated flasks containing mitomycin C inactivated mouse embryonic fibroblasts (MEFs) from e12.5 MF-1 mouse embryos in Knockout DMEM with 20% Knockout serum replacement (KO/SR) (Gibco), 4ng/mL bFGF (R&D Systems), 1% non-essential amino acids (Gibco), 1mM L-Glutamine (Gibco) and 0.1mM β-mercaptoethanol (Gibco). Cells were passaged using 1mg/mL of collagenase IV (ThermoFisher) at a split ratio of 1:3 every 3-4 days. For feeder free culture, cells were grown on rhVitronectin and E8 media (Life Technologies). These cells were passaged using ReLeSR (Stem Cell Technologies) at a ratio of 1:3-1:6 every 3-4 days. For the genetic modification work, the hESC line, Shef4 (Aflatoonian et al., 2010), was routinely grown in mTeSR1 medium (Stem Cell Technologies) on Matrigel (BD Biosciences) and passaged using collagenase IV (Stem Cell Technologies) as above.

## Embryoid bodies

To investigate population bias, cells were differentiated using embryoid bodies in the serum free, defined media, APEL (Stem Cell Technologies) (Ng et al., 2008). Single cells were sorted as described below, into the respective cell fractions and single cells were resuspended in APEL media with 10μM Y-27632 (Tocris) (Watanabe et al., 2007) at a concentration of 3000 cells/100μL. 100μL of cell suspension was pipetted into the inner 60 wells of a non-adherent U-shaped 96-well plate (Sigma) to generate EBs of 3000 cells in size. The remaining outer 36 wells were filled with 100μL of sterile PBS to humidify the plate. Plates were centrifuged at 1000rpm for 3 mins to collect cells at the bottom of the wells and EBs were left to develop for 10 days at 37°C under a humidified atmosphere of 5% $CO_2$ in air.

## Generation of a *GATA6*-GFP reporter hESC line

A *GATA6* reporter hESC line was generated using a standard gene targeting replacement vector designed to knock-in an enhanced Green Fluorescent Protein (GFP) reporter cassette by homologous recombination into exon 2 of the human *GATA6* locus at the position of the ATG translational initiation codon. The reporter cassette contained the GFP coding sequence linked via an IRES sequence to a puromycin resistance gene with a polyadenylation signal

sequence; followed by a neomycin resistance gene expressed from a constitutive promoter (PGK*neo*pA) and flanked by *frt* sites. The gene targeting vector was built utilizing recombineering technology (Zhang et al., 1998) according to standard protocols (details available on request to co-author KA) as follows. First, the reporter cassette GFP-IRES-*puro*pA/frt/PGK*neo*pA/frt was inserted directly after the start codon (ATG) of *GATA6* sequence in a BAC vector (RP11-523D21) by recombineering using the oligonucleotides hGata6-GFP-up and hGata6-GFP-dn. For making the targeting vector, a fragment comprised of 2.6 kb 5'- and 4.1 kb 3'- GATA6 homology sequences flanking the GFP cassette was subcloned from the BAC by recombineering (as previously described) into a PCR amplified p15A-HSV*tk*DTA-amp plasmid backbone using the primers hGata6-sub1 and hGata6-sub2.

hGata6-GFP-up: 5'-ACCCCACCTCAGGAGCTAGACGTCAGCTTGGAGCGGCGCCGGACCGTGGATGGTGAGCAAGGGCGAGGAGCTGTTCACCGGGGTGGTGC-3'

hGata6-GFP-dn: 5'-CCCGCGGCCCCGAAGCGCTTCGGCAAGCACCAGCCGCCGTCAGTCAAGGCATCATGACCATGATTACGCCAAGCTTGGGCTGCAGGTTCTTTCCGCCT-3'

hGata6-sub1: 5'-GAATGAGAGAGATTTTATTCAACTAAAAATAAGCAAGCTTCCTAGGTTGTGCGATCGCGATATCTTAATTAATAAGATGATCTTCTTGAG-3'

hGata6-sub2: 5'-GTTCATATACACACCCCCTCTTCGCTCCCTCCAAACAGTTATCACAACTCAGATCTTACGTATTACCAATGCTTAATCAGTGAGG-3'

The final replacement vector contained homology arms to the *GATA6* locus 5' and 3' of the desired insertion position (6.7 kb total homology), and also a negative selection marker (HSV*tk*DTA) positioned outside the homology arms. A Zinc Finger Nuclease (ZFN) was designed with specificity to the sequence of the *GATA6* gene spanning the ATG translational initiation codon in exon 2; ZFN recognition sequence; 5'AGCTTGGAGCGGCGCCGGACCGTGGATGGCCTTGACTGACGGC (ZFN cleavage site in bold; ATG codon underlined). Shef4 hESC grown in mTeSR1 medium on Matrigel were gently trypsinized and co-electroporated (800 volts, 3 µfarads) in mTeSR1 medium with 50 µg of the replacement vector (linearized at a unique restriction site) and with 25 µg of each of the two plasmids expressing the pair of ZFN polypeptides. Cells were plated onto Matrigel coated dishes and into mTeSR1 medium with ROCK inhibitor. Forty eight hours later selection was

commenced with 50 µg per ml G418. Cells were fed daily for 12 days with mTeSR1 plus G418, and then G418 resistant colonies picked into 96 well plates and into mTeSR1 medium plus G418. Ten days later cells were replica plated into 96 well plates, frozen, and genomic DNA prepared from one of the replicas. Clones with homologous integration of the vector were identified by restriction digestion of genomic DNA from the 96 well plate and Southern blotting/hybridization with appropriate flanking sequence probes. Correct gene targeted integrations were identified at a frequency of ~4% of total G418 resistant clones, and positive clones thawed and expanded. One of these clones (S4G6 4/F-9), which was heterozygous for the reporter knock-in, was further modified to excise the PGK*neo*pA gene by FLP recombination at the flanking *frt* sites in order to avoid any potential unpredicted phenotypic effects arising from the constitutively expressed PGK promoter. Excision was achieved by transient exposure of cells to cell permeable FLP recombinase (TAT-FLP) kindly donated by Frank Edenhofer and applied using an established protocol (Patsch et al., 2011). Cells were plated at clonal density and colonies screened after replica plating for sensitivity to G418. G418 sensitive colonies were further analyzed by Southern blotting/hybridization to confirm correct excision of the PGK*neo*pA selection marker via FLP recombination at the *frt* sites. One of the derivative clones obtained after FLP recombination, called S4G6 A3, and the S4G6 4/F-9 clone, were confirmed to have a normal karyotype (30 metaphases scanned), and furthermore no additional unpredicted vector integration events were detected in either of these clones by Southern blot hybridization analysis. S4G6 A3 and S4G6 4/F-9 gave essentially similar profiles when analyzed by FACS for GFP expression and SSEA3 expression.

**Antibodies**

The following monoclonal antibodies were derived from the relevant hybridomas grown in-house, pre-titered and used to detect surface antigen expression as previously described (Adewumi et al., 2007; Draper et al., 2002): MC631, anti-Stage Specific Embryonic Antigen-3 (SSEA3) (Shevinsky et al., 1982), MC813-70, anti-Stage Specific Embryonic Antigen-4 (SSEA4) (Kannagi et al., 1983), MC480, anti-Stage Specific Embryonic Antigen-1 (SSEA1) (Solter and Knowles, 1978), TRA-1-60 and TRA-1-81 (Andrews et al., 1984) and TRA-1-85 (Williams et al., 1988). Antibody P3X, from the parental myeloma, P3X63ag8 (Köhler and Milstein, 1975), was used as a negative control (Draper et al., 2002). For intra-cellular staining, commercial antibodies were obtained and used as detailed by the manufacturer as follows. OCT4A, 1:200, SOX2, 1:200 (Cell Signaling Technologies, #C52G3, #D6D9 respectively), SOX17 at 1µg/mL, GATA4 at 1µg/mL and GATA6 at 1µg/mL (R&D Systems, #AF1924, AF2606, #AF1700 respectively). Secondary antibodies were also obtained commercially and

used as per manufacturer instructions. AlexFluor647 conjugated goat-anti-mouse IgG (H+L) (Stratech #209-605-082) 1:100, AlexFluor594 conjugated donkey-anti-goat IgG+IgM (Stratech #708-585149) 1:100, AlexFluor647 conjugated donkey anti-rabbit IgG+IgM (Stratech #609-605-213) 1:100.

**Flow Cytofluorimetry and Fluorescence Activated Cell Sorting**

hESC were dissociated using trypLE (Gibco), stained for the relevant surface markers and analyzed using a BD FACS Jazz. The same machine was also used to sort cells. After sorting, single cells were seeded at clonogenic densities of 500 cells/$cm^2$ in standard hESC medium on MEF, in the presence of 10µM Y-27632 (Tocris) (Watanabe et al., 2007). Y-27632 was subsequently removed after 24h. Clonogenic assays had three technical repeats over three biological repeats. For sub-cloning experiments, single cells were sorted directly into single wells of 96-well plates in standard 20% KO/SR, DMEM and MEF conditions with 10µM Y-27632. Resulting colonies were picked initially, and then passaged using 1mg/mL collagenase IV. All sub-clones were passaged at least 5 times before analysis.

**Immunocytochemistry**

Cells were fixed using 4% paraformaldehyde for 15 minutes at room temperature, and washed twice with Dulbecco's Phosphate Buffered Saline, without calcium and magnesium (PBS). For intracellular staining, cells were permeablized and blocked in PBS with 10% fetal calf serum, 0.3mM glycine, 1% bovine serum albumin (BSA), and 0.1% Tween for 2h at room temperature. Cells were incubated with the primary antibody resuspended in blocking buffer (without 0.3mM Glycine) overnight at 4$^{o}$C, washed twice in PBS and incubated with the species specific secondary antibody for 2h at 4$^{o}$C. Finally, cells were washed twice in PBS and imaged on an InCell Analyzer 2000 (GE Healthcare). Images were analyzed using the Developer Toolbox software (GE Healthcare).

**Gene expression analysis**

Total RNA was extracted using Trizol (Life Technologies) and a centrifugation based column kit (Norgen RNA clean-up and concentration kit) followed by cDNA synthesis using the High Capacity cDNA Reverse Transcription Kit (Applied Biosystems). Quantitative real-time PCR was performed on the QuantStudio 12K Flex Real-Time PCR system (ThermoFisher) using Taqman universal master mix (Invitrogen) in conjunction with the Roche universal probe library system (Roche). Primers and probes were designed by Primer3 software (Roche) and where

possible intron-spanning primers were used. Gene expression was normalized to β-Actin in all experiments.

**Clustering, Box Plot and Kullback-Leibler Divergence Analysis**

All statistical analyses were implemented using MATLAB or GraphPad. Hierarchical clustering was performed using Spearman's rank correlation. Colormap indicates levels of expression of 1/Delta-CT values standardized by row. Box plots indicate values between the 25th and 75th percentile with level represented by the median and whisker lengths of 2.7SD. Outliers extending beyond whiskers were excluded. The Kruskal-Wallis pairwise test was used to compare distributions at a statistical significance level of $<0.05$.

Kullback-Leibler analysis was implemented in MATLAB. Kernel density estimation was used to estimate nonparametric probability density functions (PDF) from histograms. Kullback-Leibler divergence was used to quantify shape changes in the histograms. For p and q denoting two PDFs, the Kullback-Leibler symmetric divergence was calculated pairwise.

**Processing, Read Alignment and Digital Gene Expression (DGE) Matrix Construction.**

Raw sequencing data was quality filtered, adapter- and polyA-trimmed and reads satisfying a length criterion of 30nts were aligned to the human (hg19) genome using Bowtie2 (v2.2.9) with cross-species mapping reads to mouse (mm9) removed. Aligned reads were tagged with gene exons using Bedtools Intersect (v2.26.0). Digital gene expression (DGE) matrices were then generated for each time point from all Drop-seq runs. To digitally count gene transcripts, reads with the same corresponding cell barcode were aggregated together and unique UMIs and cell barcodes were merged within 1 Hamming and 2 Levenshtein distances, respectfully. We filtered all DGEs to exclude cells detecting <500 genes and <1250 transcripts from all downstream analyses.

**tSNE analysis of single cell RNA-seq data.**

DGE matrices were normalized by the number of transcripts in log space (e.g. ln(transcripts/10,000+1)). Cells were projected onto a 2D embedding using t-Distributed Stochastic Neighbor Embedding (tSNE) with cell loadings associated with 30 principal components utilizing all expressed genes as input. Cluster assignments were computed using a density-based clustering approach (DBSCAN). The degree of similarity between clusters was computed by averaging the gene expression for each cluster, and calculating a Euclidean

distance matrix between all pairs, then using this data as input for hierarchical clustering with optimal leaf ordering. We implement a negative binomial generalized linear model to identify differentially expressed genes (DEGs) enriched in each cluster. Genes satisfying an abs (log(average expression difference))>1 and P-value<0.01 were considered statistically significant. Gene Ontology (GO) enrichments were computed using all DEGs and a subset of the top 50 DEGs by average expression difference per cluster. Significant enrichments satisfying a log10(Benjamini-Hochberg(P-value)) <-3 threshold were graphed. The data discussed in this publication have been deposited in NCBI's Gene Expression Omnibus (Edgar *et al*., 2002) and are accessible through GEO series accession number GSE113168 (https://www.ncbi.nlm.nih.gov/geo/query/acc.cgi?acc=GSE113168).

## Supplementary References

Adewumi, O., Aflatoonian, B., Ahrlund-Richter, L., Amit, M., Andrews, P.W., Beighton, G., Bello, P.A., Benvenisty, N., Berry, L.S., and Bevan, S. (2007). Characterization of human embryonic stem cell lines by the International Stem Cell Initiative. Nature biotechnology *25*, 803-816.

Aflatoonian, B., Ruban, L., Shamsuddin, S., Baker, D., Andrews, P., and Moore, H. (2010). Generation of Sheffield (Shef) human embryonic stem cell lines using a microdrop culture system. In Vitro Cellular & Developmental Biology-Animal *46*, 236-241.

Andrews, P.W., Banting, G., Damjanov, I., Arnaud, D., and Avner, P. (1984). Three monoclonal antibodies defining distinct differentiation antigens associated with different high molecular weight polypeptides on the surface of human embryonal carcinoma cells. Hybridoma *3*, 347-361.

Draper, J.S., Pigott, C., Thomson, J.A., and Andrews, P.W. (2002). Surface antigens of human embryonic stem cells: changes upon differentiation in culture. Journal of anatomy *200*, 249-258.

Kannagi, R., Cochran, N.A., Ishigami, F., Hakomori, S.-i., Andrews, P., Knowles, B.B., and Solter, D. (1983). Stage-specific embryonic antigens (SSEA-3 and-4) are epitopes of a unique globo-series ganglioside isolated from human teratocarcinoma cells. The embo journal *2*, 2355.

Köhler, G., and Milstein, C. (1975). Continuous cultures of fused cells secreting antibody of predefined specificity. Nature *256*, 495-497.

Ng, E.S., Davis, R., Stanley, E.G., and Elefanty, A.G. (2008). A protocol describing the use of a recombinant protein-based, animal product-free medium (APEL) for human embryonic stem cell differentiation as spin embryoid bodies. Nature protocols *3*, 768-776.

Patsch, C., Kesseler, D., and Edenhofer, F. (2011). Genetic engineering of mammalian cells by direct delivery of FLP recombinase protein. Methods *53*, 386-393.

Shevinsky, L.H., Knowles, B.B., Damjanov, I., and Solter, D. (1982). Monoclonal antibody to murine embryos defines a stage-specific embryonic antigen expressed on mouse embryos and human teratocarcinoma cells. Cell *30*, 697-705.

Solter, D., and Knowles, B.B. (1978). Monoclonal antibody defining a stage-specific mouse embryonic antigen (SSEA-1). Proceedings of the National Academy of Sciences *75*, 5565-5569.

Watanabe, K., Ueno, M., Kamiya, D., Nishiyama, A., Matsumura, M., Wataya, T., Takahashi, J.B., Nishikawa, S., Nishikawa, S.-i., and Muguruma, K. (2007). A ROCK inhibitor permits survival of dissociated human embryonic stem cells. Nature biotechnology *25*, 681.

Williams, B., Daniels, G., Pym, B., Sheer, D., Povey, S., Okubo, Y., Andrews, P., and Goodfellow, P. (1988). Biochemical and genetic analysis of the Ok a blood group antigen. Immunogenetics *27*, 322-329.

Zhang, Y., Buchholz, F., Muyrers, J.P., and Stewart, A.F. (1998). A new logic for DNA engineering using recombination in Escherichia coli. Nature genetics *20*, 123.
